# A potential role of *p75NTR* in the regulation of circadian rhythm and incremental growth lines during tooth development

**DOI:** 10.3389/fphys.2022.981311

**Published:** 2022-09-23

**Authors:** Hongyan Yuan, Bo Xie, Xia Yu, Cheng Lin, Meng Li, Yixin Zhang, Xuqiang Zou, Mingjie Lu, Manzhu Zhao, Xiujie Wen

**Affiliations:** ^1^ Department of Orthodontics, The Affiliated Stomatology Hospital of Southwest Medical University, Luzhou, China; ^2^ Department of Oral Maxillofacial Surgery, Shenzhen Hospital, Southern Medical University, Shenzhen, China; ^3^ Chongqing Key Laboratory for Oral Diseases and Biomedical Sciences, Chongqing Municipal Key Laboratory of Oral Biomedical Engineering of Higher Education, College of Stomatology, Chongqing Medical University, Chongqing, China

**Keywords:** p75 neurotrophin receptor, circadian rhythm, mineralization, cell polarity, tooth development

## Abstract

**Objective:** Tooth morphogenesis and the formation of hard tissues have been reported to be closely related to circadian rhythms. This study investigates the spatiotemporal expression and relationship of *p75NTR* with core clock genes, mineralization-related or odontogenesis-related genes, and aims to derive the potential role of *p75NTR* in regulating circadian rhythm and incrementality growth line formation during tooth development.

**Materials and methods:** The dynamic morphology of the rat dental germ was observed at seven stages (E14.5 d, E16.5 d, E18.5 d, P.N. 4 d, P.N. 7 d, P.N. 10 d, and P.N. 15 d). Next, the expressions of *p75NTR* and other target factors were traced. The ectomesenchymal stem cells (EMSCs) were isolated from the E18.5d rat dental germs and synchronized using 50% of fetal bovine serum. Then, they were cultured in light/light (L.L.), dark/dark (D.D.), and light/dark (L.D.) conditions for 48 h. The total RNA was collected every 4 h, and the circadian rhythm dynamics of target factors were observed. To reveal the mechanism further, *p75NTR* was down-regulated in *p75NTR* ^*ExIII−/−*^ mice and up-regulated in immortalized mouse dental apical papilla progenitor cells. The change tendencies of other target factors were also detected.

**Results:** The clock genes *Bmal1*, *Clock*, *Per1*, and *Per2* were all expressed in tooth germs before the formation of dental hard tissues and demonstrated a regular oscillating expression pattern in EMSCs from dental germs. Their expression was affected by the L.D. stimulus, and most of them were promoted by D.D. conditions. *p75NTR* presented a similar expression pattern and a positive or negative relationship with most clock genes, mineralization-related and odontogenesis-related factors, such as brain and muscle ARNT-like protein-1 (*Bmal1*), runt-related transcription factor 2 (*Runx2*), alkaline phosphatase (*ALP*), MSH-like 1 (*MSX1*), dentin matrix acidic phosphoprotein 1 (*Dmp1*), and dentin sialophosphoprotein (*Dspp*). Moreover, the arrangement, morphology, and even boundary in pre-odontoblast/pre-ameloblast layers were disordered in the *p75NTR* ^*ExIII−/−*^ mice.

**Conclusion:** Circadian rhythm was found to affect tooth development. *p75NTR* might play a crucial role in regulating clock genes in the mineralization and formation of the dental hard tissues. *p75NTR* is actively involved in the odontoblast-ameloblast junction and cell polarity establishment during tooth morphogenesis.

## Introduction

Most physiological and behavioral processes, such as hormone secretion, metabolism, growth, sleep, among others, are governed by circadian rhythms, which are managed by internal biological clocks (Richards and Gumz, 2013; Neumann et al., 2019). The suprachiasmatic nucleus (SCN), located in the brain’s anterior hypothalamus, is generally considered the “master clock” controlling the circadian rhythms (Weaver, 1998). Moreover, peripheral clocks have been discovered in several body tissues and found to be regulated by the SCN *via* a transcriptional-translational feedback loop (TTFL) (Okawa et al., 2020; Allada and Bass, 2021). Importantly, circadian locomotor output cycles kaput (*Clock*) and brain and muscle ARNT-like protein-1 (*Bmal1*) serve as positive feedback signals, form heterodimers, and activate the transcription of period (*Per*) and cryptochrome (*Cry*) genes by binding to a cis-regulatory enhancer sequence known as the E-box element on the target gene promoter (Gekakis et al., 1998; Shearman et al., 2000). After reaching a certain concentration, *Per* and *Cry* proteins are phosphorylated and translocated into the nucleus to inhibit the transcriptional activation of Clock/Bmal1 by competitively binding to the E-box element (Brown et al., 2012). This negative TTFL acts as a core circadian regulator to maintain the 24-h rhythm.

Clock genes play an essential role in tooth development (Nirvani et al., 2017; Papakyrikos et al., 2020). *Clock*, *Bmal1*, *Per*, and *Cry* are expressed in dental tissues, especially in dental hard tissues during tooth development (Zheng et al., 2011; Lacruz et al., 2012). The phenomenon of regular incremental growth lines (e.g., daily Retzius’s lines in enamel, von Ebner’s lines in dentine) implies that the formation of dental hard tissues characterizes the circadian rhythm and is tightly controlled by the time (Antoine et al., 2009; Lacruz et al., 2012). The collagen production in dentin follows a 12 hour-pattern, with twice as much collagen secreted during the 12 h of daylight than the 12 h of nighttime (Lopez Franco et al., 2006). Previous studies have demonstrated that tooth morphogenesis and hard tissue formation have been closely related to circadian rhythms (Lacruz et al., 2012). However, how the core clock genes affect tooth development is still unclear.

p75 neurotrophin receptor (*p75NTR*) demonstrated a strong expression in epithelial-mesenchymal interaction, dental papilla, and dental follicle during tooth development, and positively regulated the mineralization in ectomesenchymal stem cells (EMSCs) (Wen et al., 2012; Li et al., 2017). Further research revealed that the incisors’ daily mineralization speed and the incremental growth line width were significantly lower in *p75NTR* knockout mice than in wild-type mice (Wang et al., 2020; Zhao et al., 2020). There is also evidence that *p75NTR* regulates tooth morphogenesis and mineralization along with the circadian rhythm and incremental growth line formation during tooth development. *p75NTR*, a member of the tumor necrosis factor receptor superfamily, has been reported to manage a wide range of biological functions via multiple intracellular signaling pathways. Recently, *p75NTR* was reported to be controlled by *Clock/Bmal1* by bonding to the E-box element and was considered a clock gene–regulating oscillatory component of circadian rhythms (Baeza-Raja et al., 2013). Therefore, *p75NTR* might act as a clock-controlled gene in tooth development and regulate periodic mineralization during incremental growth line formation.

The aims of this study are to investigate the spatiotemporal expression and relationship of *p75NTR* with core clock genes, mineralization-related or odontogenesis-related genes, and further to reveal the potential role of *p75NTR* in regulating the circadian rhythms and incremental growth line formation during tooth development via the *in vivo* experiment of *p75NTR* ^*ExIII*^ knockout mice and *in vitro* experiment of EMSCs cultured under light/light (L.L.), dark/dark (D.D.), and light/dark (L.D.) conditions, which would contribute to illustrate the circadian rhythm and biomineralization in tooth development.

## Materials and methods

### Experimental animals

Sprague–Dawley (S.D.) rats were provided by the Chongqing Key Laboratory of Oral Diseases and Biomedical Sciences, Chongqing Medical University. *p75NTR* knockout mice used in this study were gifted by the Jackson Laboratory (#:031,162). In 1992, it was reported that these mutant mice have a targeted deletion of exon III of the *p75NTR* (*p75NTR* ^*ExIII−/−*^) and could not express functional full-length *p75NTR* (Lee et al., 1992). The presence of a vaginal plug is considered embryonic day 0.5 (E 0.5 d), and the day of littermate birth is regarded as post-natal day 1(PN1 d). All procedures were approved by the Medical Ethics Committee of the Chongqing Medical University.

### H.E. and immunohistochemistry staining

**FIGURE 1:**
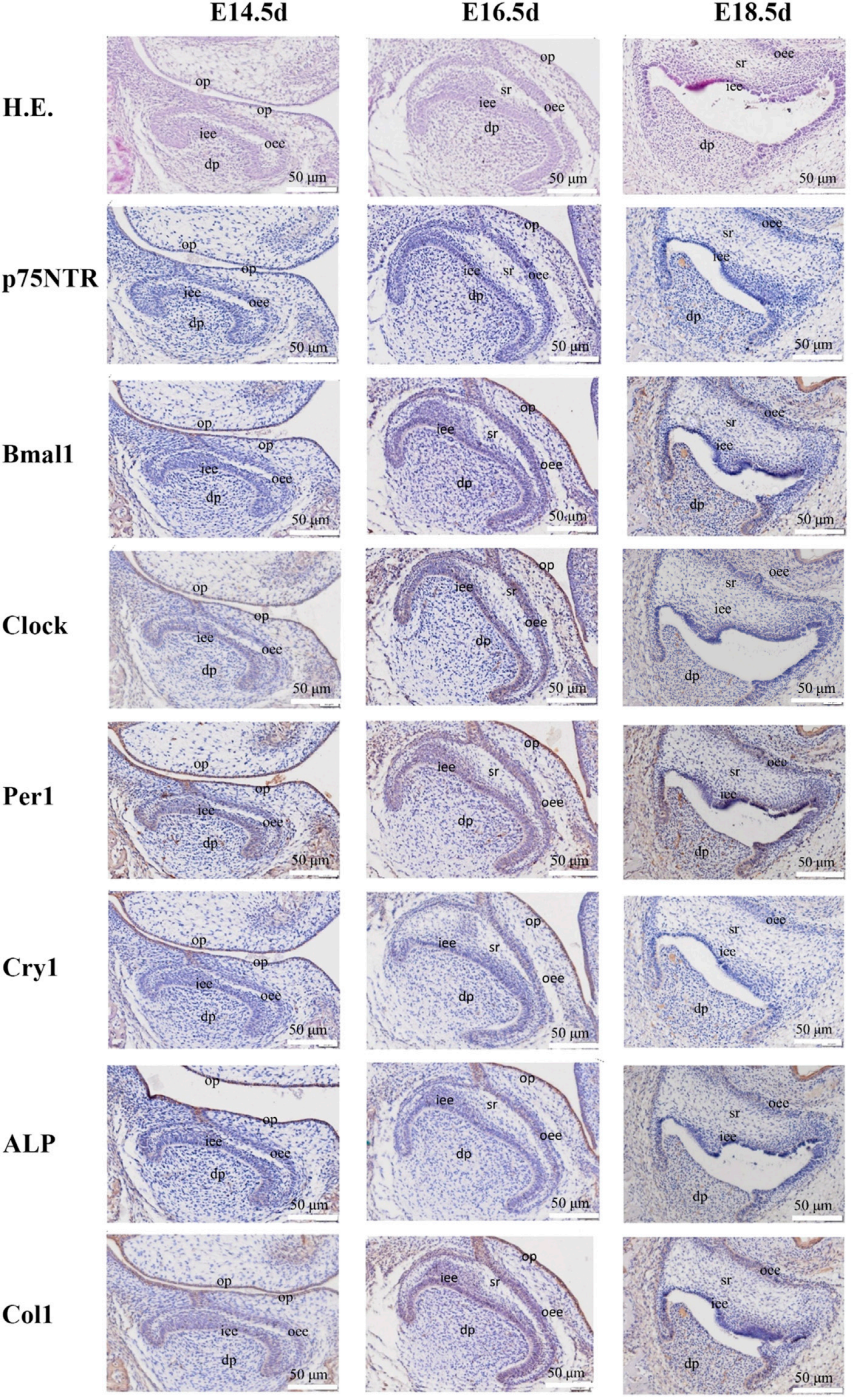
The images of H.E. staining and immunohistochemistry staining for prenatal rat dental germs. H.E. staining demonstrated that the rat dental germs entered the early cap stage at E14.5 d, the cap stage and early bell stage at E16.5 d, and the bell stage at E18.5 d. The separation between the pre-odontoblast and pre-ameloblast layers occurred in all E18.5 d species. Immunohistochemistry staining revealed that *Clock*, *Per1*, and *Col1* were detected in the epithelial-mesenchymal interaction area, dental follicle, and dental papilla at E14.5 d, and became stronger at E16.5 d when *p75NTR*, *Bmal1*, and *ALP* were detected. All the factors were expressed at E18.5 d, but *Cry1* showed the weakest expression. All experiments were repeated three times independently. o*p*: oral epithelium; dp: dental papilla; iee: inner enamel epithelium; oee: outer enamel epithelium; sr: stellate reticulum. The scale bar represents 50 μm, respectively.

**FIGURE 2:**
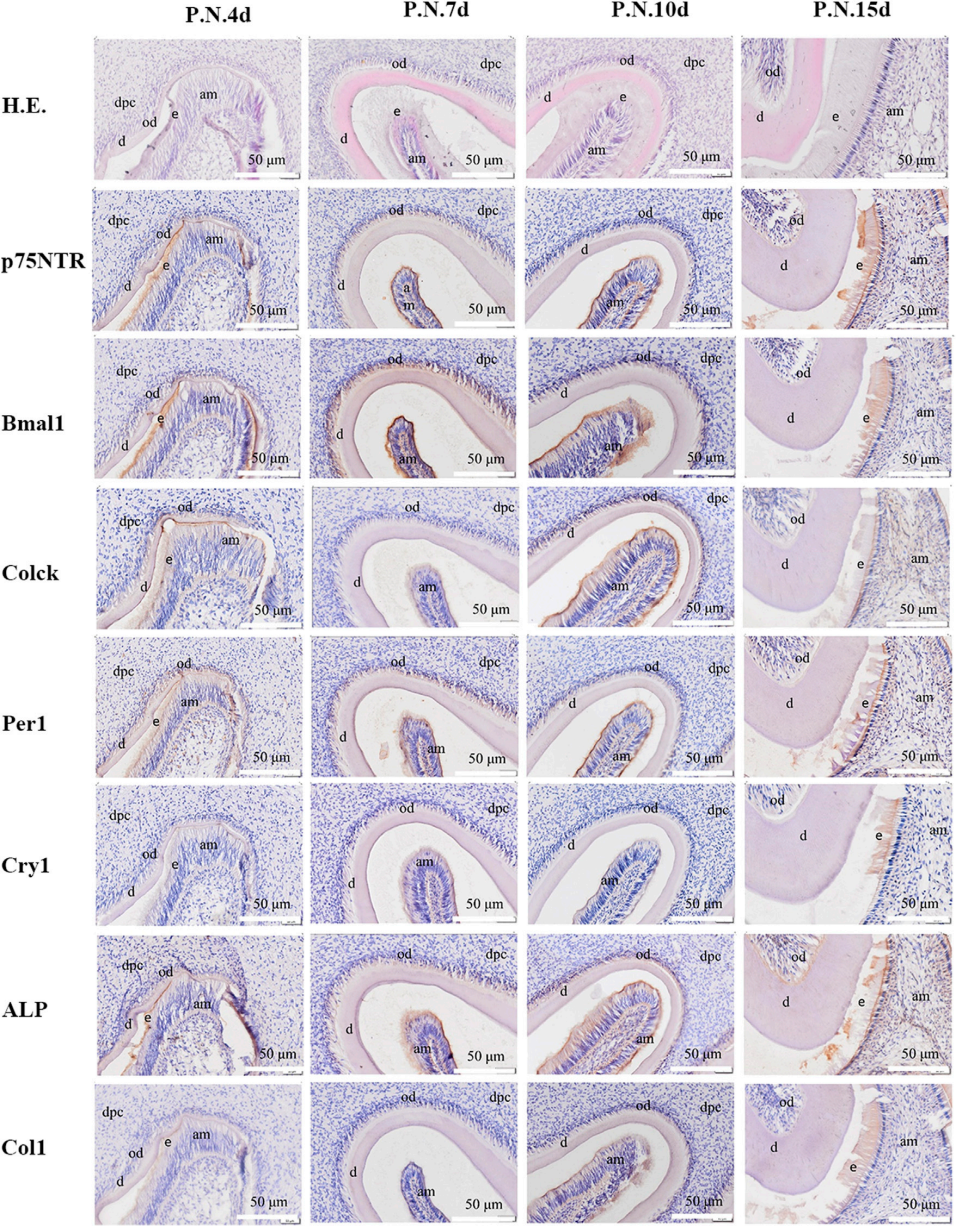
The images of H.E. staining and immunohistochemistry staining for post-natal rat dental germs. H.E. staining revealed that the morphogenesis of molar cusps was completed, and hard tissues (enamel and dentine) began to form at PN4 d. The hard tissues gradually thickened at PN7 d. The tooth roots began to shape at PN10 d and PN15 d. The separation continued between the inner enamel epithelium and enamel in all the post-natal species. Immunohistochemistry staining showed that all the factors were detected and distributed in odontoblast and ameloblast layers, and then in dental papilla in the post-natal species. All experiments were repeated three times independently. dpc: dental papilla cells; od: odontoblast; am: ameloblast; e: enamel; d: dentin. The scale bar represents 500 and 50 μm, respectively.

The first molars were dissected from the E14.5 d, E16.5 d, E18.5 d, P.N.4 d, P.N.7 d, P.N.10 d, and P.N.15 d rats (Figures 1, 2) and the E16.5d *p75NTR* ^*ExIII−/−*^ and *p75NTR* ^*ExIII+/+*^ mice (Figure 4), fixed in 4% paraformaldehyde, decalcified with 10% EDTA, and embedded in paraffin. The 6-μm sections of tissue specimens were obtained for H.E. and immunohistochemistry staining. The primary antibodies were used in this study are as follows: rabbit anti-rat *p75NTR* (1:1,500; Abcam, Cambridge, MA, United States, ab245134, monoclonal), rabbit anti-rat *BMAL1* (1:1,000; Abcam, Cambridge, MA, United States, ab230822, monoclonal), rabbit anti-rat *CLOCK* (1:1,000; Abcam, Cambridge, MA, United States, ab3517, polyclonal), rabbit anti-rat *PER1* (1:500; Bioss, Beijing, China,bs-2350R, polyclonal), rabbit anti-rat *CRY1* (1:500; Bioss, Beijing, China bs-11441R, polyclonal), rabbit anti-rat *ALP* (1:500; Bioss, Beijing, China, bs-2928R, polyclonal), rabbit anti-rat *COL1* (1:500; Bioss, Beijing, China, bs-0578R, polyclonal). These specimens were treated with the DAB Detection Kit Streptavidin-Biotin (ZSGB-BIO, Beijing, China) and Hematoxylin and Eosin Staining Kit (Beyotime, Shanghai, China) according to the manufacturer’s protocols, followed by visualization under phase-contrast microscopy.

### Isolation and culture of EMSCs from dental germs

The first molars of the upper and lower jaws were dissected from seven to ten embryos of E18.5d rats. The minced tissue was mixed with 1% trypsin/1 mM of the EDTA solution (Sigma, St. Louis, MO, United States) at 37°C for 10 min and neutralized with Dulbecco’s modified Eagle’s medium/Ham’s F12 (DMEM/F12) (Sigma, St. Louis, MO, United States) containing 10% of FBS (Gibco Waltham, MA United States). Next, the suspension was centrifuged at 800 rpm for 5 min. The cell pellet was resuspended in DMEM/F12 supplemented with 10% FBS and antibiotics (100 μg/ml of penicillin and 100 μg/ml of streptomycin) at a density of 2 * 10^5^/ml and then cultured at 37°C in a 5% CO2 humidified incubator. The culture medium was changed every 2 days and cells were passaged when the cell density was fused to 70 %–80%. E18.5 d rat EMSCs at passage three were used in the following experiment.

### Clock synchronization experiment of EMSCs under three conditions

E18.5 d rat EMSCs were seeded into 6-well plates at a density of 1 × 10^5^ cells per well and incubated in the routine medium as mentioned above until confluency. They were synchronized using 50% of the horse serum (Gibco Waltham, MA,United States), and total RNA was collected every 4 hours over a circadian cycle at the following zeitgeber time (Z.T.) after synchronization: ZT0, 4, 8, 12, 16, 20, 24, 28, 32, 36, 40, 44, and 48. The cells were exposed to the serum for 2 h and then incubated under three conditions, namely L.L.-cycle, D.D.-cycle, and 12-h L.D.-cycle (12 h dark and 12 h light), until sample collection.

### Genotype identification of *p75NTR*-knockout mice

All mice were housed under specific pathogen-free conditions (22°C, 12/12-h L.D., 50%–55% humidity) in the Chongqing Key Laboratory for Oral Diseases and Biomedical Sciences. The *p75NTR* knockout (*p75NTR* ^*ExIII−/−*^) and wild-type (*p75NTR* ^*ExIII+/+*^) littermates used in this research were obtained from mating between heterozygous (*p75NTR* ^*ExIII+/−*^) females and males. The tail DNA was extracted and used to determine their genotypes by PCR analysis (Zhang et al., 2015).

### Wild-type mice feeding under D.D. And 12-h D.L. conditions

*p75NTR* ^*ExIII+/+*^ mice were fed and mated under D.D.-cycle and D.L.-cycle (natural day and night), respectively, at PN7 d, four wild-type mice were killed at 7:30 a.m. and 7:30 p.m. The maxilla and mandible of five mice were separated under sterile conditions, and the first molars of mice were removed under the anatomical microscope and then divided into enamel organs and dental papilla. All experiments were repeated at least three times independently. Total RNA was extracted from the isolated dental papilla with the steady pure universal RNA Extraction Kit II and subsequently analyzed quantitatively by RT qPCR.

### *p75NTR* over-expression plasmid constructs and transfection

The coding region of *p75NTR* was amplified from the cDNAs of mice and then cloned into vector GV492. The plasmid GV492-*p75NTR* was co-transfected with lentivirus helper plasmids (Helper 1.0 and Helper 2.0) into HEK-293T cells using Lipofectamine 2000 (Invitrogen, United States) according to the manufacturer’s protocol, the negative control carried corresponding fluorescent markers and contain resistant genes, but do not express other target genes. Supernatants containing the virus were collected 72 h following transfection and then infected the immortalized mouse dental apical papilla progenitor cells. iSCAP (Using the previously characterized reversible immortalization system, which expresses SV40 T antigenflanked with Cre/loxP sites, Wang et al. (2014) demonstrated that the mouse SCAPs can be effectively immortalized with an enhanced proliferative activity) was gifted by Chongqing Key Laboratory of Oral Diseases and Biomedical Sciences. The cells were selected with 2 *μg*/ml of puromycin 48 h later. *p75NTR* over-expression iSCAP and negative control iSCAP were seeded into 6-well plates at a density of 1 × 10^5^ cells per well and incubated in the routine medium until confluency. All experiments were repeated at least three times independently.

### Quantitative RT qPCR

According to the manufacturer’s specifications, the total RNA was extracted using Steady Pure Universal RNA Extraction Kit II. The total RNA was determined using nanodrop spectroscopy before cDNA synthesis using Evo M-MLV Mix Kit with gDNA Clean for qPCR, with Oligo dT at 37°C for 15 min in a 20-μL reaction. Real-time RT-PCR was conducted on 0.01ug per well cDNA samples with SYBR Green PCR SuperMix (Biorad) using the CFX Connect™ Real-Time PCR Detection System (BioRad) under the following cycling conditions: 95°C for 3 min; 40 cycles of 95°C for 5 s; and 60°C for 30 s. Results were normalized relative to a housekeeping gene’s GAPDH expression. Primers were designed against the following genes: glyceraldehyde 3-phosphate dehydrogenase (*GAPDH*), low-affinity neurotrophin receptor *p75NTR* (*p75NTR*), MAGE family member D1 (*Mage-D1*), aryl hydrocarbon receptor nuclear translocator-like (*ARNTL or Bmal1*), clock circadian regulator (*Clock*), period circadian clock 1 (*Per1*), period circadian clock 2 (*Per2*), runt-related transcription factor 2 (*Runx2*), alkaline phosphatase (*ALP*), collagen type I (*Col1*), Msh Homeobox 1 (*Msx1*), distal-less homeobox 1 (*Dlx1*), dentin matrix acidic phosphoprotein 1 (*DMP1*) and dentin sialophosphoprotein (*Dspp*) (Tables 1, 2 enlist the primer sequences used in this study). The specificity of all primers was tested by BLAST, and the melting curve of the RT-PCR result again proved its specificity.

**TABLE 1:** Rat oligonucleotide primers used in this study.

Genes	Upstream (5′–3′)	Downstream (5′–3′)	Product Size (bp)
*GAPDH*	AAG​TTC​AAC​GGC​ACA​GTC​AAG​G	ACG​CCA​GTA​GAC​TCC​ACG​ACA​T	140
*p75NTR*	CCT​CAT​TCC​TGT​CTA​TTG​CTC​CA	GCG​CCT​TGT​TTA​TTT​TGT​TTG​C	105
*Bmal1*	CAG​AAG​CAA​ACT​ACA​AGC​CAA​C	CGG​TCA​CAT​CCT​ACG​ACA​AAC	100
*Clock*	CAG​TTC​TTA​CAG​ACA​TCT​CGG​TTG	AAA​GTG​CTC​TGT​TGT​AGT​GGA​AAG	89
*Per1*	ATG​CAC​TTT​CAG​GCT​CCA​GT	TTG​CTT​GTA​TGG​CTG​CTC​TG	187
*Runx2*	GAA​CCA​AGA​AGG​CAC​AGA​CAG​AA	GGC​GGG​ACA​CCT​ACT​CTC​ATA​CT	113
*Dlx1*	CAG​CCC​CTA​CAT​CAG​TTC​CG	CTT​CTC​CGC​CTT​CCA​CCA​C	116

**TABLE 2:** Mice oligonucleotide primers used in this study.

Genes	Upstream (5′–3′)	Downstream (5′–3′)	Product Size (bp)
*GAPDH*	TGA​CCT​CAA​CTA​CAT​GGT​CTA​CA	CTT​CCC​ATT​CTC​GGC​CTT​G	85
*p75NTR*	CCA​GAG​CGA​GAC​CTC​ATA​GC	AGA​TGG​AGC​AAT​AGA​CAG​GAA​TG	121
*Mage-D1*	ACT​TAC​GAC​CCT​CAC​CTA​ATC​TG	ATG​GGC​ACC​TTC​GTG​TAG​TC	154
*Bmal1*	AAG​ACA​ATG​AGC​CAG​ACA​ACG	TCC​CAT​CTA​TTG​CGT​GTC​G	147
*Clock*	CCA​TCC​AGT​ATG​CCA​CAG​AAC	TCA​CCA​CCT​GAC​CCA​TAA​GC	172
*Per1*	CCA​GTA​CAA​CCA​AGC​GTA​AAT​G	TTG​CTG​ACG​ACG​GAT​CTT​TC	123
*Per2*	AGC​GGC​TTA​GAT​TCT​TTC​ACT​C	TCT​CAT​TCT​CGT​GGT​GTT​TCC	88
*Runx2*	TTC​CAG​ACC​AGC​AGC​ACT​CC	GCT​TCC​GTC​AGC​GTC​AAC​AC	189
*Col1*	TAA​GGG​TCC​CCA​ATG​GTG​AGA	GGG​TCC​CTC​GAC​TCC​TAC​AT	170
*ALP*	TGA​CTA​CCA​CTC​GGG​TGA​ACC	TCT​GGT​GGC​ATC​TCG​TTA​TCC	94
*Msx1*	TCC​TCA​AGC​TGC​CAG​AAG​ATG	CTT​GCG​GTT​GGT​CTT​GTG​C	155
*Dlx1*	ATG​CCA​GAA​AGT​CTC​AAC​AGC	GAA​GGA​GAC​ATT​TGC​TGG​TTG	83
*DMP1*	CAG​AGG​GAC​AGG​CAA​ATA​GTG	CAT​CGC​CAA​AGG​TAT​CAT​CTC	168
*Dspp*	GGA​CAC​AGC​AGG​ATA​GGT​AGC​AG	CAC​TTT​CGT​CAC​TTC​CGT​TAG​ATT​C	124

### Statistical analyses

Following the determination of normal distribution by F-test, normally distributed data were analyzed by *t* test and non-normally distributed data were analyzed by Mann-Whitney test. All the data are expressed as the means ± standard deviations (S.D.). Statistical significance was assessed using the Prism 8.0 software (GraphPad Software, San Diego, CA, United States). *p*-values ＜ 0.05 were considered statistically significant. All experiments were repeated thrice.

## Results

### Dynamic histological observation of rat tooth development

H.E. staining demonstrated that the rat dental germs entered the early cap stage at E14.5 d, the cap stage and early bell stage at E16.5 d, and the bell stage at E18.5 d (Figure 1 and Supplementary Figure S1). In the post-natal species, the morphogenesis of molar cusps was completed, and hard tissues (enamel and dentine) began to be detected at PN4 d (Figure 2 and Supplementary Figure S2). Next, the hard tissues gradually thickened at PN7 d. The tooth roots began to shape at PN10 d and PN15 d. Interestingly, the inner enamel epithelium was easy to separate from odontoblast layers in the E18.5 d species during tissue sectioning, indicating that the adhesion between odontoblasts and ameloblasts would weaken at E18.5 d. This separation between the inner enamel epithelium and enamel continued to occur in all the species in the following four post-natal stages.

Immunohistochemistry staining illustrated that *Clock*, *Per1*, and *Col1* were expressed in the epithelial-mesenchymal interaction area, dental follicle, and dental papilla at E14.5 d (Figure 1 and Supplementary Figure S1). The other *p75NTR*, *Bmal1*, *Cry1*, and *ALP* factors were either little or no expressed. At E16.5 d, the expressions of Clock, Per1, and Col1 became stronger, and the expressions of *p75NTR*, *Bmal1*, and *ALP* were detected. All the factors were expressed at E18.5 d, but *Cry1* showed the weakest expression. In the post-natal species (Figure 2 and Supplementary Figure S2), all the factors were detected and distributed in the odontoblast and ameloblast layers, and further in the dental papilla. Of the eight factors detected in this assay, *Bmal1* showed the strongest expression, *Per1*, *Clock*, and *p75NTR* demonstrated a moderate expression, *ALP* and *Col1* had an increased expression, and *Cry1* had the weakest expression throughout.

### *In vitro* observation of the circadian rhythm dynamics in rat EMSCs

**FIGURE 3:**
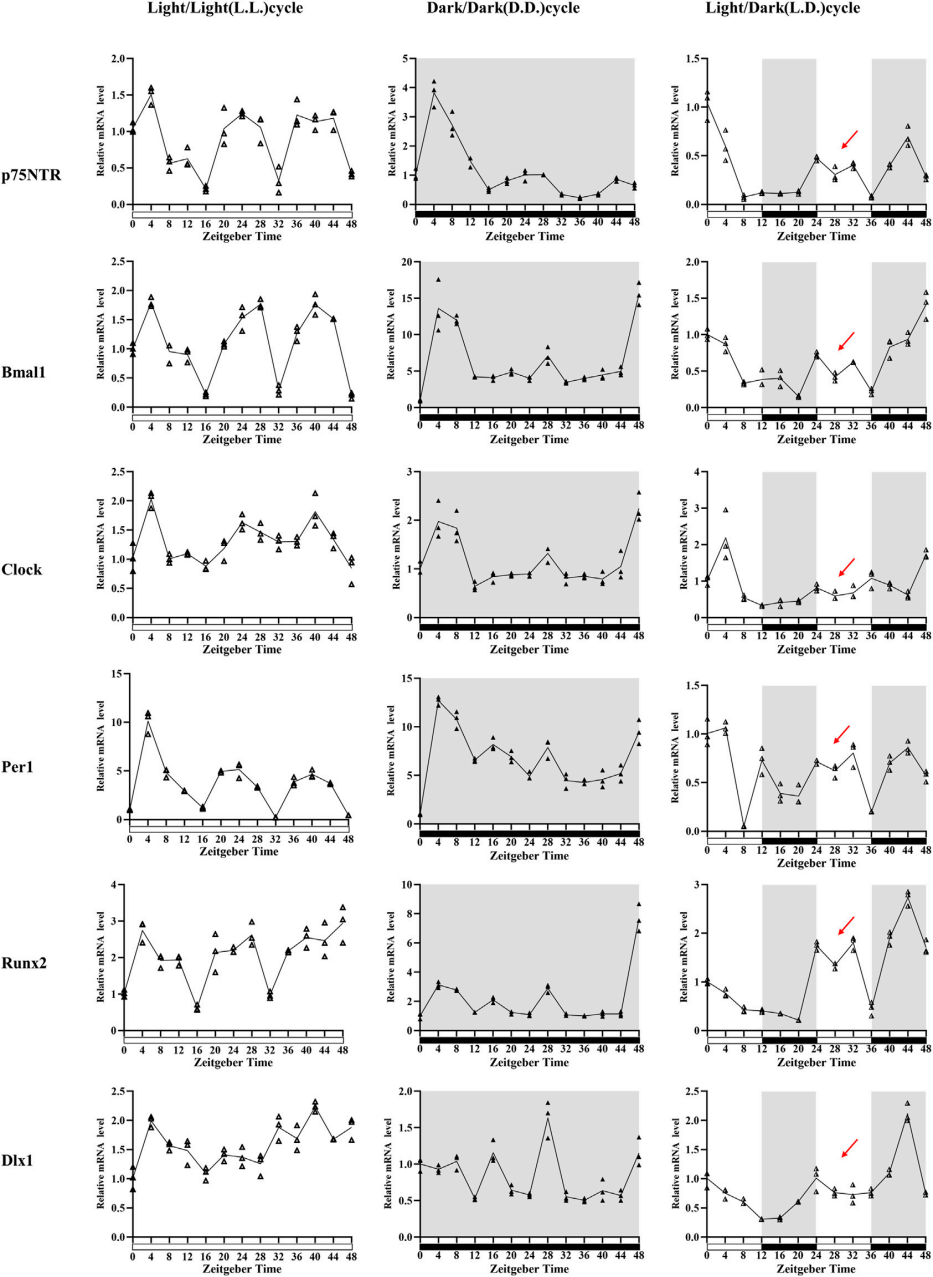
The *in vitro* observation of circadian rhythm dynamics in rat E18.5 d EMSCs. Quantitative RT-PCR analyses depicted that *p75NTR* mRNA expression patterns were similar in three conditions: higher at about ZT4, ZT24, and ZT36-44 and lower at about ZT16, ZT32, and ZT48. However, the patterns had highest amplitudes, and a supramaximal peak was detected at ZT4 in the D.D. condition. Of the three clock genes, *Bmal1* and *Clock* showed a similar mRNA expression pattern to *p75NTR* in three conditions. *Per1* was similar in the L.L. condition, but four, not three, peak times presented in the L.D. and D.D. conditions. The mineralization-related factors *Runx2* also showed a similar mRNA expression pattern to *p75NTR* in L.L. and D.D. conditions, but four peak times were observed in the L.D. condition: ZT0, ZT24, ZT32, and ZT44. The odontogenesis-related factor *Dlx1* showed irregular oscillating expressions: ZT4, ZT20, ZT32, and ZT40 in the L.L. condition; ZT8, ZT16, ZT28, ZT40, and ZT40 in the D.D. condition; and ZT0, ZT24, and ZT44 in the L.D. condition. Besides, the peak at ZT28 in all the six detected factors in D.D. condition, but it was replaced by a V-shape (as indicated by the red arrows) in the L.D. condition when the cells were exposed to the second 12-h light stimulus. All experiments were repeated three times independently. Data are expressed as mean ± S.D. *n* = 13 per group.

Quantitative RT-PCR analyses demonstrated the temporal expression profiles of *p75NTR*, clock, mineralization-related and odontogenesis-related genes in E18.5 d EMSCs collected every 4 h during the ZT0 h–ZT48 h following serum synchronization (Figure 3). The relative expression levels of *p75NTR* mRNA were significantly higher at ZT4, ZT24, and ZT36-44 and were lower at ZT16, ZT32, and ZT48 in the L.L. condition. The peak times of *p75NTR* mRNA expression in the D.D. and L.D. conditions were similar to that in the L.L. condition. Still, the amplitudes in the D.D. condition were the biggest (the *Y*-axis unit in D.D. is more than twice or three times that in the L.L. and L.D. conditions). A supramaximal peak at ZT4 was also detected. The results revealed that the mRNA expression of *p75NTR* characterized the prominent oscillation in a day and was affected by light stimulus.

Of the three clock genes detected in this study, *Bmal1* and *Clock* depicted a similar mRNA expression pattern and tendency to *p75NTR* in the three conditions, indicating a possible relation of *p75NTR* with *Bmal1* and *Clock* in tooth development. The *Per1* mRNA expression pattern in the L.L. condition was similar to that of *p75NTR* and showed the three peak times. However, four peak times were observed in the other two conditions. Besides, *Bmal1* amplitudes were considerably higher in the D.D. condition (the *Y*-axis maximum unit is 20), whereas *Per1* amplitudes were considerably higher in L.L. and D.D. conditions (the *Y*-axis maximum unit is 15). These results indicated that light stimulus would play a crucial role in the temporal expression and oscillation of clock genes in rat EMSCs.

The mineralization-related factor *Runx2* revealed the three mRNA expression peaks at ZT4, ZT28, and ZT48 in both L.L. and D.D. conditions, which were similar to that of *p75NTR*, *Bmal1*, and *Clock*, indicating a circadian rhythm oscillation in rat EMSCs. However, four peak times were observed in the L.D. condition: ZT0, ZT24, ZT32, and ZT44. The peak times of odontogenesis-related factor *Dlx1* showed irregular oscillating expressions, namely ZT4, ZT20, ZT32, and ZT40 in the L.L. condition; ZT8, ZT16, ZT28, ZT40, and ZT40 in the D.D. condition; and ZT0, ZT24, and ZT44 in the L.D. condition.

There are two variables in this experiment: light stimulus and time. The D.D. condition considerably promoted the mRNA expression of *Bmal1* and *Runx2*, then *p75NTR*. In contrast, the L.L. condition significantly promoted the mRNA expression of *Per1*. Interestingly, a peak was found at ZT28 in all the six detected factors under the D.D. condition, but this peak became a V-shape (as indicated by the red arrows in Figure 3) under the L.D. condition when the cells were exposed to the second 12-h light stimulus. The results suggested that light stimulus affected the mRNA expression oscillation of rat EMSCs. The peak-to-peak periods of *p75NTR*, *Bmal1*, *Clock*, and *Runx2* were maintained for about 24 h, and the peak times were nearly consistent between L.L. and D.D. conditions.

### Identification of *p75NTR* knockout mice and the histological observation of dental germs

**FIGURE 4:**
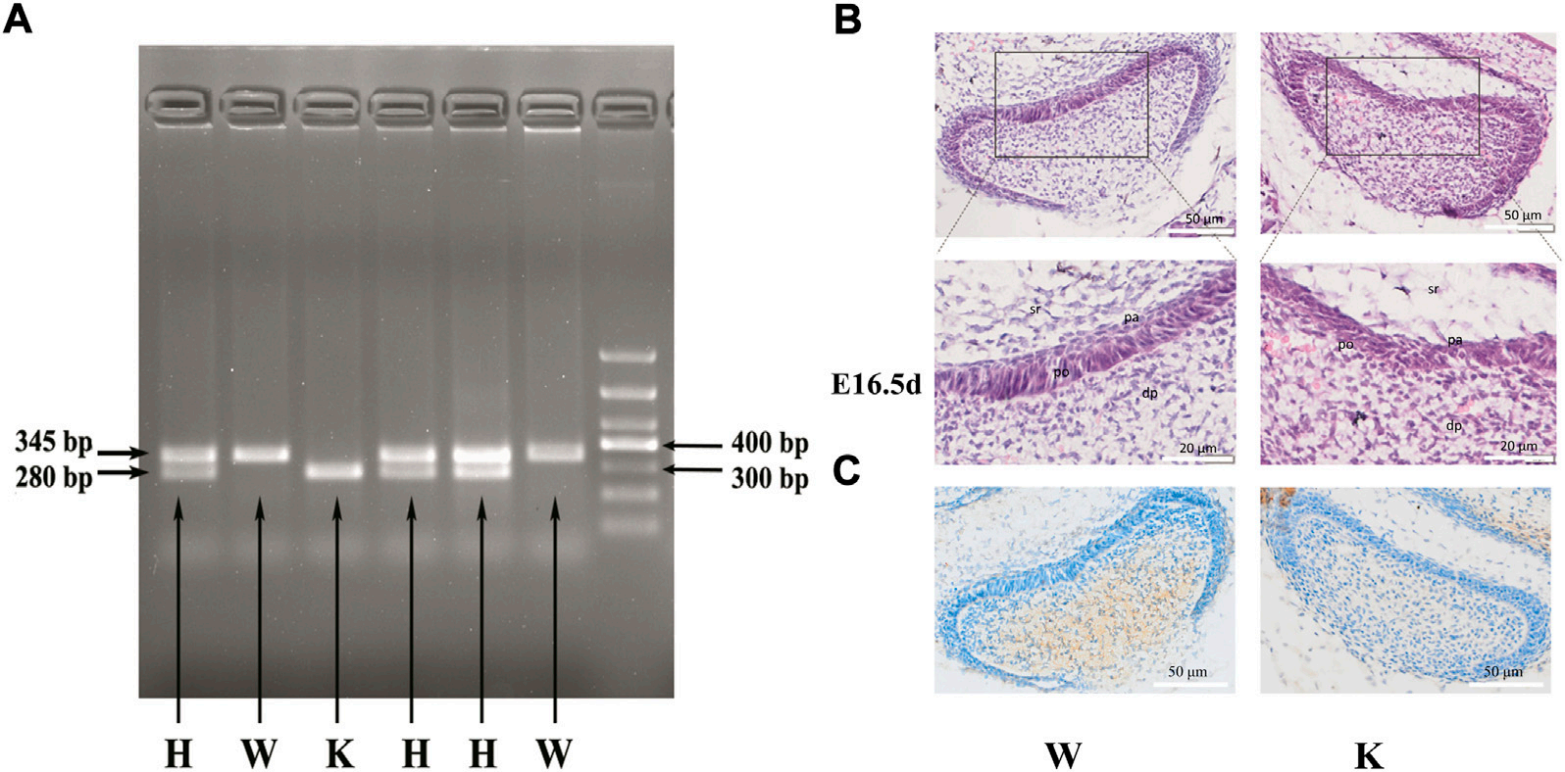
The genotype identification and dental germ morphology of p75NTR knockout mice. Of the six littermates detected, three with two bands of 280 bp and 345 bp were identified as heterozygous mice, two with one band of 345 bp were identified as wild-type mice, and one with one band of 280 bp was identified as knockout mice **(A)**. H.E. staining revealed that the pre-odontoblast and pre-ameloblast layers tightly adhered to each other in the E16.5 d mouse dental germ **(B)**. The long columnar pre-odontoblasts and short columnar pre-ameloblasts were regularly arranged with apparent polarity and boundary in the wild-type mice. In contrast, the regular shape, polarity, and boundary disappeared in the knockout mice. **(C)** Immunohistochemical staining was performed on the wild-type mice and *p75NTR* knockout mice. The results showed that *p75NTR* was almost not expressed in the tooth germs of the knockout mice. dp: dental papilla; po: pre-odontoblast; pa: pre-ameloblast; sr: stellate reticulum. The scale bar represents 50 and 20 μm, respectively. Wild-type mice, *n* = 3; *p75NTR* knockout mice, *n* = 2.

The genotypes of mice were determined by RT-PCR (Figure 4A). Of the six detected littermates, three with two bands of 280 bp and 345 bp were identified as heterozygous mice, two with one band of 345 bp were identified as wild-type mice, and one with one band of 280 bp was identified as the knockout mice. Immunohistochemical staining was performed on the wild-type mice and *p75NTR* knockout mice to further confirm the knockout of *p75NTR*. The results showed that *p75NTR* was almost not expressed in the tooth germs of the knockout mice (Figure 4C).

H.E. staining revealed that the dental germs of E16.5 d mouse were present at the early bell stage (Figure 4B). The pre-odontoblast and pre-ameloblast layers tightly adhered to each other in wild-type and knockout mice. Interestingly, long columnar pre-odontoblasts and short columnar pre-ameloblasts in wild-type mice were regularly arranged with apparent polarity and boundary. In contrast, the pre-odontoblast and pre-ameloblast layers in the knockout mice were not observed their regular shape and polarity. The boundary between pre-odontoblast and pre-ameloblast layers also disappeared. The results indicated that *p75NTR* might play a critical role in the shape and the polarity of odontoblasts and ameloblasts during tooth development.

### *In vivo* observation of circadian rhythm dynamics in the dental papilla of the model mice

**FIGURE 5:**
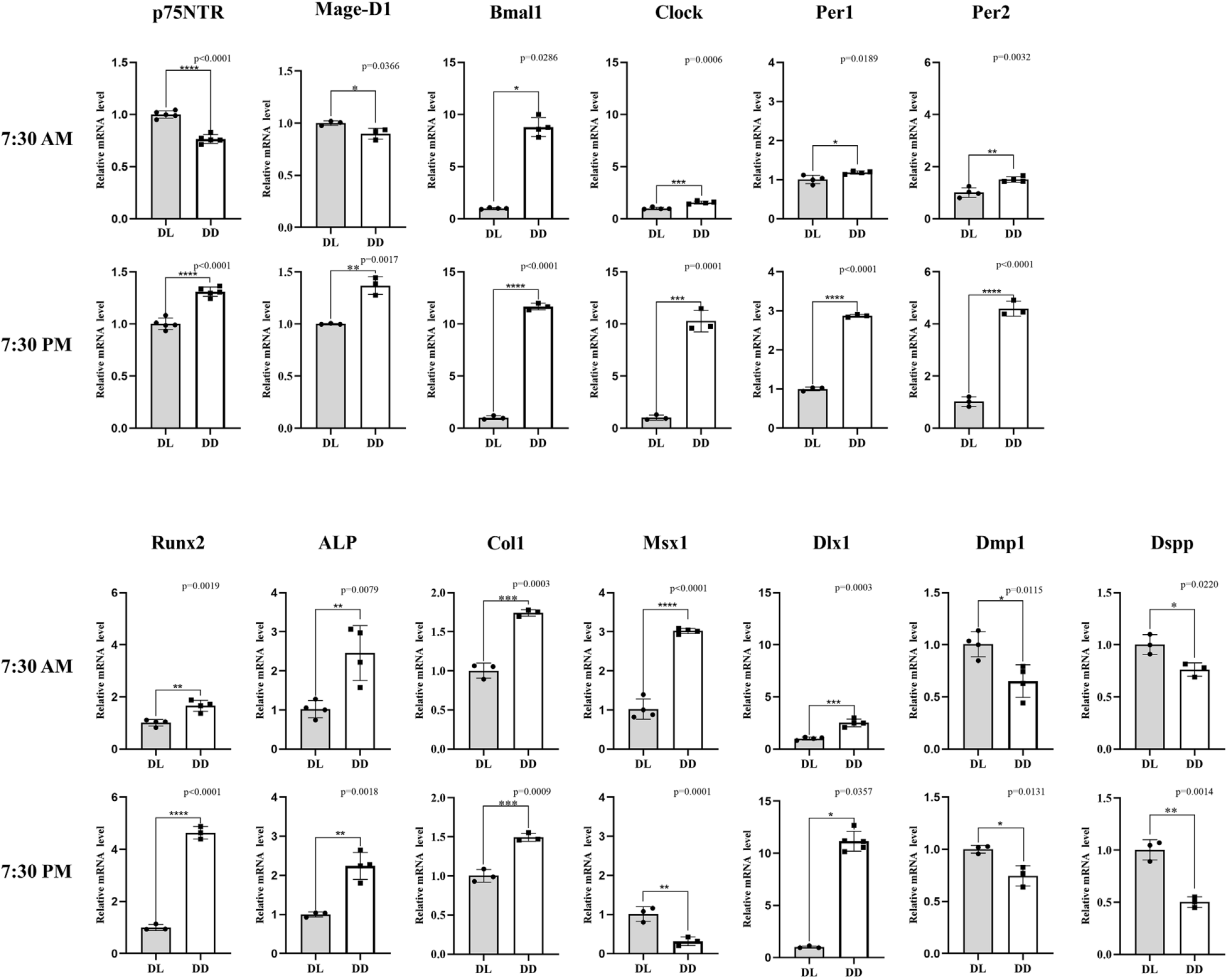
The *in vivo* observation of circadian rhythm dynamics in PN7 d dental germs of wild-type mice. *p75NTR* mRNA showed a significant difference between D.D. and L.D. conditions (*p* < 0.05), but the change tendencies at two sampling times were opposite: *p75NTR* mRNA expression in the L.D. condition was significantly higher at 7:30 a.m. and significantly lower at 7:30 p.m. in the D.D. condition (*p* < 0.05). The expression pattern of Mage-D1 was similar to that of p75NTR. Most factors (*Bmal1*, *Clock*, *Per1*, *Per2*, *Runx2*, *ALP*, *Col1*, and *Dlx1*) showed the same change tendency at the two sampling times: mRNA expression was significantly higher in the D.D. condition than that in the L.D. condition (*p* < 0.05). The change amplitudes of *Clock*, *Per1*, *Per2*, *Runx2*, and *Dlx1* were significantly larger at the sampling time of 7:30 PM. Contrary to *p75NTR*, *Msx1* mRNA expression in the L.D. condition was significantly lower at 7:30 a.m. and significantly higher at 7:30 p.m. compared with the D.D. condition (*p* < 0.05). *Dmp1*and *Dspp* mRNA expression in the D.D. condition was significantly lower than that in L.D. condition at both sampling times (*p* < 0.05). All experiments were repeated at least three times independently. Data are expressed as mean ± S.D. L. D. Condition group, *n* = 11; D. D. Condition group, *n* = 5.

The results of PN7 d wild-type mice indicated that the gene expression of dental papilla was not only related to D.D. and L.D. conditions, but also related to sampling time (Figure 5). *p75NTR* mRNA presented a significant difference between D.D. and L.D. conditions (*p* < 0.05). However, the change tendencies at two sampling times were reversed. *p75NTR* mRNA expression in the L.D. condition was significantly higher at 7:30 a.m. and significantly lower at 7:30 p.m. than in the D.D. condition (*p* < 0.05). The results demonstrated that *p75NTR* mRNA expression fluctuated in a day and was significantly affected by light stimulus, further confirming that *p75NTR* might be involved in regulating the circadian rhythms of dental papilla. The expression pattern of *Mage-D1* was similar to that of *p75NTR*. Unlike *p75NTR* and *Mage-D1*, all the four clock genes were significantly more expressed in the D.D. condition than in the L.D. condition (*p* < 0.05). Except for *Bmal1*, which showed the same big change at both two sampling times of 7:30 a.m. and 7:30 p.m., the changes of *Clock*, *Per1*, and *Per2* were small at 7:30 a.m. and much larger at 7:30 p.m. The results demonstrated that the clock gene expression in dental papilla fluctuated in a day and greatly increased under the D.D. condition.

The mineralization-related factors of *Runx2* ,*ALP,* and *Col1* revealed the same tendency that their mRNA expressions under the D.D. condition were significantly higher than that under the L.D. condition (*p* < 0.05), but the change of *Runx2* between 7:30 a.m. and 7:30 p.m. was obviously bigger. All the factors of *Msx1*, *Dlx1*, *Dmp1* and *Dspp*, which have been recognized as the key factors related to tooth development, revealed a significant mRNA expression between D.D. and L.D. conditions (*p* < 0.05), but the change tendencies were inconsistent. The *Dlx1* mRNA expression pattern was similar to that of *Clock*, *Per1*, *Per2*, and *Runx2*. Notably, the same tendency that the *Dlx1* mRNA expression under the D.D. condition was higher than that under the L.D. condition was shown at both sampling times, but the change was much more prominent at 7:30 p.m.. In contrast to *Dlx1*, *Dmp1,* and *Dspp* showed a reverse tendency that its mRNA expression under the D.D. condition was lower than that under the L.D. condition at both sampling times (*p* < 0.05). Unlike all the factors detected in this assay, the *Msx1* mRNA expression in the L.D. condition was significantly lower at 7:30 a.m. but significantly higher at 7:30 p.m. than under the D.D. condition. This expression pattern was quite the opposite of *p75NTR*, indicating that *p75NTR* might be negatively related to *Msx1* in the circadian rhythm of tooth development. Most of the mineralization-related and odontogenesis-related factors detected in this study revealed similar expression patterns to core clock genes, implying that the circadian rhythm would greatly affect the formation of dental hard tissues. These might be the possible reason for the formation of incremental growth lines, such as daily Retzius’s lines in enamel and von Ebner’s lines in dentine.

### Effect of *p75NTR* knockout or over-expression on tooth circadian rhythm and mineralization

**FIGURE 6:**
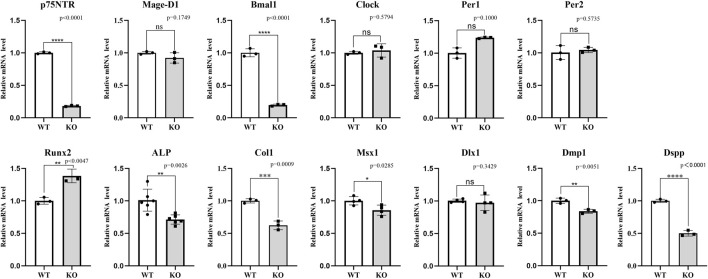
The *in vivo* assays for the effect of *p75NTR* knockout on the mRNA expression in dental germs at PN7d. As expected, *p75NTR* mRNA in the knockout mice was significantly lower than in THE wild-type mice (*p* < 0.05). *Mage-D1*, *Bmal1*, *ALP*, *Col1*, *Msx1*, *Dmp1*, and *Dspp* showed the same change in the *p75NTR* knockout mice, indicating a positive relationship with *p75NTR*. In contrast, *Runx2* showed the reverse change, presenting a negative relationship with *p75NTR*. *Clock*, *Per1*, *Per2*, and *Dlx1* showed no significant difference between *p75NTR* knockout and wild-type mice (*p* > 0.05). All experiments were repeated at least three times independently. Data are expressed as mean ± S.D. L. D. Condition group, *n* = 6; D. D. Condition group, *n* = 6.

The effect of *p75NTR* knockout was determined by the *in vivo* experiment of the model mice. Figure 6 shows the mRNA expression difference between knockout and wild-type mice. As expected, *p75NTR* mRNA in the knockout mice was significantly lower than in wild-type mice (*p* < 0.05). However, the mRNA expression of *Mage-D1* was not significantly reduced in the knockout mice (*p* > 0.05). Interestingly, of the four core clock genes detected, *Bmal1* demonstrated the exact noticeable change and tendency as *p75NTR*, which indicated that *Bmal1* mRNA expression might be closely related to *p75NTR*. The other three clock genes depicted the reverse tendency. *Per1* mRNA was statistically higher in the knockout mice (*p* > 0.05). *Clock* and *Per2* mRNA were slightly higher in the knockout mice but showed no significant difference (*p* > 0.05). Of the three mineralization-related factors, *Runx2* mRNA expression in the knockout mice was significantly higher than in the wild-type mice (*p* < 0.05). In comparison, *ALP* and *Col1* mRNA expression in the knockout mice was significantly lower than in the wild-type mice. *Dlx1* showed no significant difference among the four odontogenesis-related factors (*p* > 0.05). *Msx1*, *Dmp1*, and *Dspp* were significantly lower in the knockout mice than in the wild-type mice (*p* < 0.05).

**FIGURE 7:**
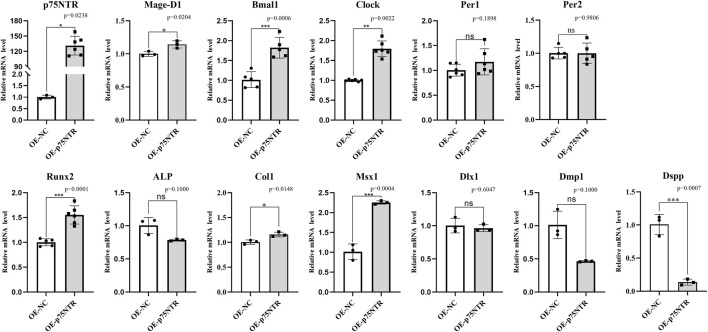
The *in vitro* assays for the effect of *p75NTR* over-expression on the mRNA expression in immortalizing stem cells from dental apical papilla (iSCAP). *p75NTR* was expected to be significantly higher expressed in the over-expression group than that in the control group (*p* < 0.05). *Mage-D1*, *Bmal1*, *Clock*, *Runx2*, *Col1*, and *Msx1* showed an apparent positive relationship when *p75NTR* was significantly up-regulated. In contrast, *ALP*, *Dmp1*, and *Dspp* showed a negative relationship with *p75NTR*. *Per1*, *Per2*, *ALP*, *Dlx1*, and *Dmp1* showed no significant difference between the over-expression group and control group (*p* > 0.05) with lower than that in wild-type mice (*p* < 0.05). All experiments were repeated at least three times independently. Data are expressed as mean ± S.D. Overexpression negative control group, *n* = 3; p75NTR over-expression group, *n* = 3.

The effect of *p75NTR* over-expression was determined by the *in vitro* experiment of iSCAP. Figure 7 shows that *p75NTR* mRNA expression was considerably higher in the over-expression group than in the control group (*p* < 0.05). Seven factors presented a significant change (*p* < 0.05): six positive relationships (*Mage-D1*, *Bmal1*, *Clock*, *Runx2*, *Col1*, and *Msx1*) and one negative relationships (*Dspp*) with p*75NTR*. The other five factors (*Per1*, *Per2*, *ALP*, *Dlx1*, and *Dmp1*) showed no significant change (*p* > 0.05). Compared with the results presented above, the change tendency of *Bmal1*, *Per1*, *Per2*, *Col1, Msx1*, and *Dlx1* with *p75NTR* knockout and over-expression were consistent in the *in vivo* model mice and *in vitro* cell experiment. However, the change tendency of *Runx2* and *Dspp* was reversed. The *Mage-D1* and *Clock* showed no significant difference in the model mice experiment but exhibited a positive relationship with *p75NTR* over-expression in the cells experiment.

## Discussion

Teeth are an essential model for gaining insights into the general processes of biomineralization (Jussila and Thesleff, 2012; Bakhit et al., 2018; Pagella et al., 2020). It comprises three mineralized tissues, namely dentine, cementum, and enamel, of which the biomineralization is specific for each dental hard tissue. Moreover, they are unique in the body, involving specific proteins, such as collagen and non-collagenic matrix proteins, not found elsewhere. Therefore, the mechanism of tooth biomineralization is still unclear. The evidence exhibited regular incremental growth lines in all three dental hard tissues mentioned above, indicating that tooth biomineralization was controlled by time and is a characteristic of circadian rhythm (Zheng et al., 2011; He et al., 2019; Papakyrikos et al., 2020). In this study, the clock genes were detected to be expressed in dental germs during tooth development, and *p75NTR* was found to play an essential role in regulating the circadian rhythm during the formation of dental hard tissues. In order to reveal the mechanism of *p75NTR* in regulating clock genes in the mineralization and formation of the dental hard tissues, we used four models in this study. The model of rats was used to observe the dynamic morphogenesis of the first molar and the expression of *p75NTR and* clock factor protein. The model of EMSCs were used to explore the possible oscillation relationship between *p75NTR* and core clock factors. The *p75NTR* knockout mouse is a model for downregulation of *p75NTR* expression, and overexpression of *p75NTR* in iSCAP cells is a model for upregulation of *p75NTR* expression.

The tooth morphogenesis is triggered by the sequential and reciprocal interactions between ectomesenchyme generated from the cranial neural crest and dental epithelium (Jussila and Thesleff, 2012; Bakhit et al., 2018). The dynamic histological observation of this study revealed that the rat molar germs entered the early cap stage at E14.5 d, the cap stage and early bell stage at E16.5 d, and the bell stage at E18.5 d. The morphogenesis of molar cusps was completed at PN4 d. These results were consistent with that of previous studies (Taniguchi et al., 1999; Yang et al., 2020). Interestingly, the separations between pre-odontoblast and pre-ameloblast layers were found to occur in all the E18.5 d rat first molar, implying a significant decrease in the cell adhesion between odontoblasts and ameloblasts at this stage. This finding indicated that the cell crosstalk of epithelial-mesenchymal interactions might primarily occur during the early tooth morphogenesis. In the post-natal rat first molar, the dental hard tissues began to be detected at PN4 d, and the inner enamel epithelium was also found easy to separate from enamel. It might be the structure of dentine tubules that enhanced the adhesion of odontoblasts to dentine.

The clock genes of *Bmal1*, *Clock*, *Per1*, and *Cry1* were detected in this study. Dynamic histological observation demonstrated that *Clock* and *Per1* were apparently expressed in the rat dental germs at E14.5 d, *Bmal1*, and *Cry1* began to be represented at E16.5 d. Although *Cry1* was weakly expressed throughout, the other three displayed an increased expression in the following rat first molar germs. The expression’s initial times were largely consistent with the report by Zheng et al. [12] that *Bmal1*, *Clock*, *Per1*, and *Per2* were not detected at E14 d or 15 d and began to be expressed in the mouse dental germs at E17 d. The expression distributions were completely consistent with the previous studies (Polly, 2015; Allada and Bass, 2021). The expressions of *Bmal1*, *Clock*, *Per1*, and *Cry1* were initially detected in the pre-odontoblast and pre-ameloblast layers and then detected in the dental papilla cells, stratum intermedium, and stellate reticulum. These results indicated that the clock genes might participate in the complex epithelial-mesenchymal crosstalk networks and mainly regulate dental hard tissue formation during tooth development (Tao et al., 2016; Pincha et al., 2022).

*p75NTR*, a well-conserved transmembrane neurotrophin receptor, was shown to play a critical role in tooth morphogenesis and mineralization in the previous studies by this study’s researchers (Wen et al., 2012; Li et al., 2017; Wang et al., 2020; Zhao et al., 2020). Baeza-Raja et al. (2013) reported that *p75NTR* expression oscillated *via* the direct binding of *Clock/Bmal1* to noncanonical E-box elements present in the *p75NTR* promoter. *p75NTR* might be a novel clock gene–regulating oscillatory components of circadian rhythms. Therefore, *p75NTR* was selected and investigated in this study to determine its potential role in the circadian rhythm and incremental growth line formation during tooth development. The dynamic expression of *p75NTR* was similar to that of clock genes during the development of rat dental germs, indicating its relationship with the circadian rhythm. Moreover, *p75NTR* knockout mice exhibited the disorder of pre-odontoblast/pre-ameloblast arrangement and morphology shape and even the disappearance of boundaries between pre-odontoblast and pre-ameloblast layers. There is another possibility that these phenotypes are delayed because *p75NTR* is knocked out. In general, this finding indicated that *p75NTR* might regulate cell polarity during tooth development. Cell polarity is critical in cellular processes ranging from cell migration to asymmetric cell division and axon and dendrite specification (Piroli et al., 2019). *p75NTR* was reported to directly interact with the polarity protein Par-3 and recruited to regulate the axon-glial junction, forming a complex that points to a critical role in establishing the cell polarity for myelination (Chan et al., 2006). As a transmembrane receptor, *p75NTR* might participate in the odontoblast-ameloblast junction and cell polarity establishment during tooth morphogenesis.

The ablation of SCN was reported to result in a disrupted patterning of rat’s incremental lines in dentine and supported the involvement of the circadian clock in tooth development (Ohtsuka-Isoya et al., 2001). The opinion was confirmed by the report that *Bmal1* ^*−/−*^ mice showed the fainter daily lines in dentine than *Bmal1* ^*+/+*^ and *Bmal1* ^*+/−*^ mice (He et al., 2019). The phenomenon of incremental lines might be related to the disorder of pre-odontoblast/pre-ameloblast polarity and the arrangement found in *p75NTR* ^*ExIII−/−*^ mice of this study. *p75NTR* was speculated to play a crucial role in regulating clock genes during the formation of dental hard tissues. The recent research certainly supported this speculation that the incisors’ daily mineralization speed and incremental growth line width were significantly lower in *p75NTR* ^*ExIII−/−*^ mice than in *p75NTR* ^*ExIII+/+*^ mice (Wang et al., 2020; Zhao et al., 2020). Importantly, further studies are needed to reveal the signaling networks under this process.

Previous studies reported the detection of clock genes in dental germs and depicted a regular oscillation expression pattern, indicating that biological clocks affect tooth development (Nirvani et al., 2017; Papakyrikos et al., 2020; Huang et al., 2021). The 48-h circadian rhythm dynamics in rat EMSCs showed that the clock genes *Bmal1*, *Clock*, and *Per1* presented a regular oscillation in mRNA expression. A similar oscillation was observed in *p75NTR* and *Runx2* mRNA expression but not in *Dlx1* mRNA expression, indicating that *p75NTR* and mineralization-related factor *Runx2* might be involved in the circadian rhythm of tooth development. Previous studies support this finding that clock genes (*Bmal1*, Clo*ck*, *Per1*, and *Per2*) and two markers of ameloblast differentiation (amelogenin and kallikrein-related peptidase 4) have regular oscillations in ameloblasts (Zheng et al., 2013; Huang et al., 2021). Moreover, this oscillation was affected by L.D. stimulus. The D.D. condition significantly increased the mRNA expression of *Bmal1*, *p75NTR*, and *Runx2* in this study. The light stimulus disturbed the oscillation peaks of all the detected factors. These results were confirmed by the *in vivo* experiment in this study. The clock genes (*Bmal1*, *Clock*, *Per1*, and *Per2*), mineralization-related factors (*Runx2*, *ALP*, and *Col1*), and odontogenesis-related (*Dlx1*) in dental germs presented a similar tendency that the mRNA expression was considerably higher in the D.D. condition than in L.D. Moreover, this change was much more prominent in most of them at sampling times of 7:30 p.m. These results indicated that the D.D. condition promoted the dental mineralization, which was consistent with the previous reports that the mineralization of dental hard tissues increased in the night during the day (Lacruz et al., 2012; Satou et al., 2019).

The data in this study further confirm that circadian rhythms are involved in tooth development. However, the molecular mechanisms of the effects of clock genes in tooth development and incremental growth lines are unclear. *p75NTR* was reported to directly bond to the *Clock/Bmal1* heterodimer via the E-box element and participated in regulating circadian rhythms (Baeza-Raja et al., 2013). In a recent study by Zhao et al. (2020) the calcein fluorescence assay showed that the distance between the calcein fluorescence bands was significantly lower than that in wild-type and heterozygous mice, indicating that *p75NTR* would regulate the daily mineralization speed and incremental growth line width during tooth development. It was also proposed that *p75NTR* might participate in regulating circadian rhythm during dental incremental line formation and *Mage-D1* might play an underlying role in this process. In this study, *Mage-D1* expression was positively correlated with *p75NTR* except that *Mage-D1* was not significantly reduced with *p75NTR* knockout, implying that *Mage-D1* expression is not completely regulated by *p75NTR*. Wang et al. (2020) believed that *p75NTR* can also regulate tooth mineralization via enhancing the *PI3K/Akt/β-catenin* pathway, further illustrating that *Mage-D1* is not the only bridge factor for *p75NTR* to regulate tooth periodic development and rhythmic mineralization. To further reveal its effects and mechanisms in circadian rhythms during tooth development, *p75NTR* knockout or over-expression was performed in this study. *Bmal1* and *Msx1* showed a positive relationship with *p75NTR* in both *in vivo* model mice and *in vitro* cell experiments. *Per1*, *Per2*, and *Dlx1* showed no significant change when *p75NTR* was up- and down-expressed. In the cell experiment, the *Mage-D1* and *Clock* was positively related with *p75NTR* but uncorrelated with *p75NTR* in the model mice. Interestingly, *Runx2* was positively related to *p75NTR* in the cell experiment and negatively related in the model mice. This contradictory conclusion is attributed to the diverse biological functions of *p75NTR* and complex signal regulation mechanism. Because of the formation of bone tissue, another process of mineral deposition *in vivo*, our previous research showed that the mineralized development of mouse femur was inhibited after *p75NTR* knock (Zhao et al., 2020). However, Wang et al. (2020) found that loss of *P75NTR* upregulated *Runx2* expression, thereby promoting BMSCs mineralization *in vitro*. Thus, the signaling network related to the regulation of biological mineralization by *p75NTR* is more complex than initially understood, and further research is still needed in the future. In contrast, *ALP*, *Dmp1*, and *Dspp* were negatively related to *p75NTR* in the cell experiment and in model mice. These reverse tendencies might be the different cell niches between *in vitro* cell experiments and *in vivo* model mice. The data in this study indicated that *p75NTR* might participate in regulating circadian rhythms during tooth development via clock genes *Bmal1* and *Clock*, especially in mineralization and dental hard tissue formation. This is consistent with the previous studies that the clock gene *Bmal1* was involved in the up-regulation of mineralization in mouse bone marrow stromal cells (Chen et al., 2012; Samsa et al., 2016). Fu et al. (2005) and Min et al. (2016) also reported that *Bmal1* promoted the expression of mineralization-related factors *Runx2* and *OCN*, which was inhibited by *Per1/Per2* and *Cry1/Cry2*. Therefore, it was speculated that the core circadian regulator of negative TTFL was involved in cell mineralization, and *p75NTR* might be crucial in this process.

## Conclusion

The clock genes *Bmal1*, *Clock*, *Per1*, and *Per2* were all detected in tooth germs before the formation of dental hard tissues and showed a regular oscillating expression pattern in EMSCs from dental germs. Their expression was affected by L.D. stimulus, and most of them were promoted by D.D. conditions. *p75NTR* showed a close relationship with majority of the clock genes, mineralization-related and odontogenesis-related factors detected in this study, such as *Bmal1*, *Runx2*, *ALP*, *Msx1*, and *Dmp1*. It was speculated that *p75NTR* might participate in cell mineralization and dental hard tissue formation *via* the clock genes *Bmal1* and *Clock*. Moreover, *p75NTR* plays a potential role in the odontoblast-ameloblast junction and cell polarity establishment during tooth morphogenesis. Of course, the limitations are also present in this study. Some results in the *in vitro* experiment did not match and were even contradictory to those in the *in vivo* model mice. For example, *Runx2* was positively related to *p75NTR* in the *in vivo* experiment and negatively related to *p75NTR* in the *in vivo* model mice. The *in vivo* cell niche and the signaling networks regulating tooth development are more complex than initially understood. Further studies are still needed in the future.

## Data Availability

The raw data supporting the conclusion of this article will be made available by the authors, without undue reservation.
