# Supplementary material for: A potential role of p75NTR in the regulation of circadian rhythm and incremental growth lines during tooth development
Source: Front Physiol. 2022 Sep 23;13:981311. doi: 10.3389/fphys.2022.981311 (PMC9539461; doi:10.3389/fphys.2022.981311)

E14.5d

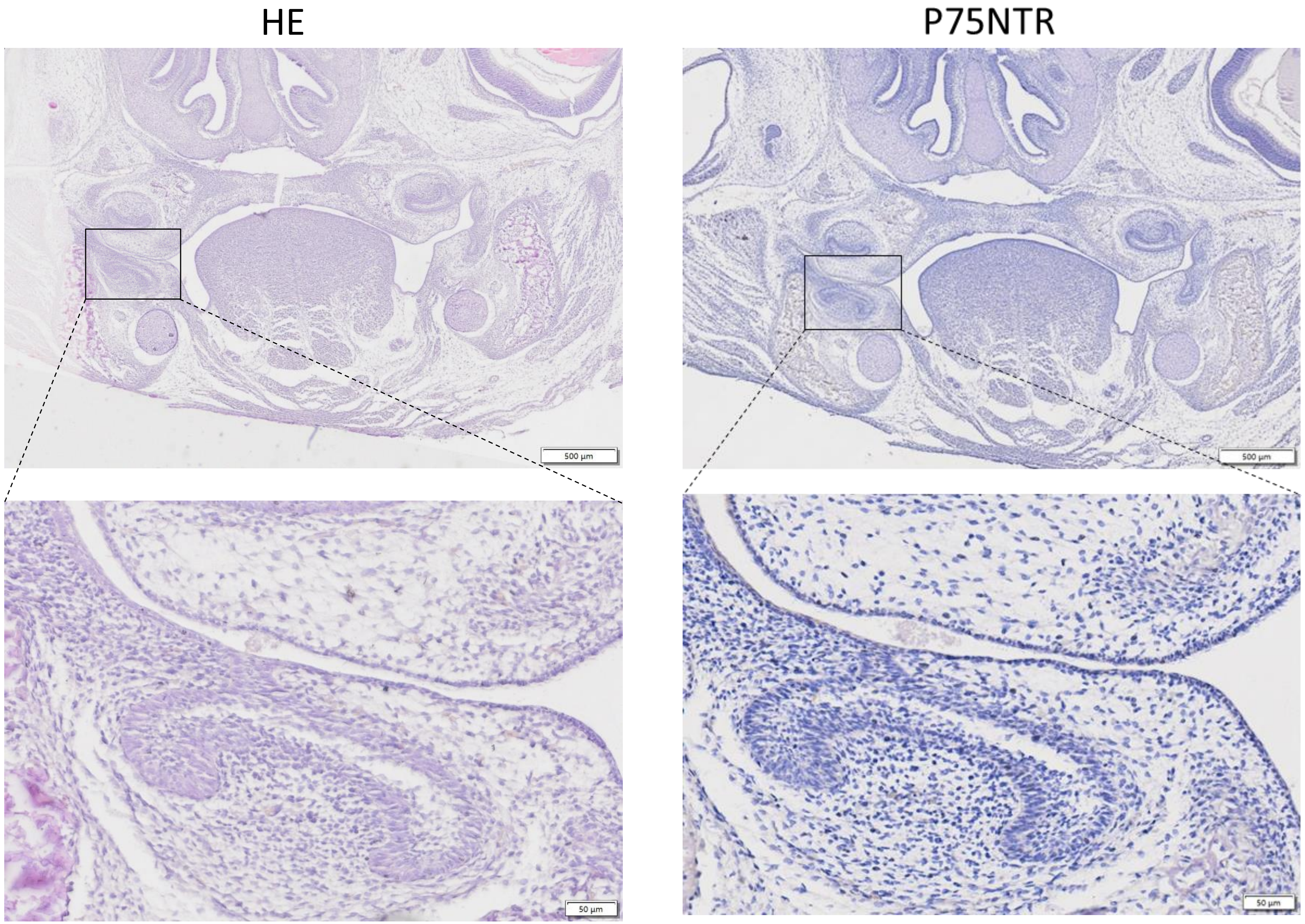

E14.5d

Bmal1 clock

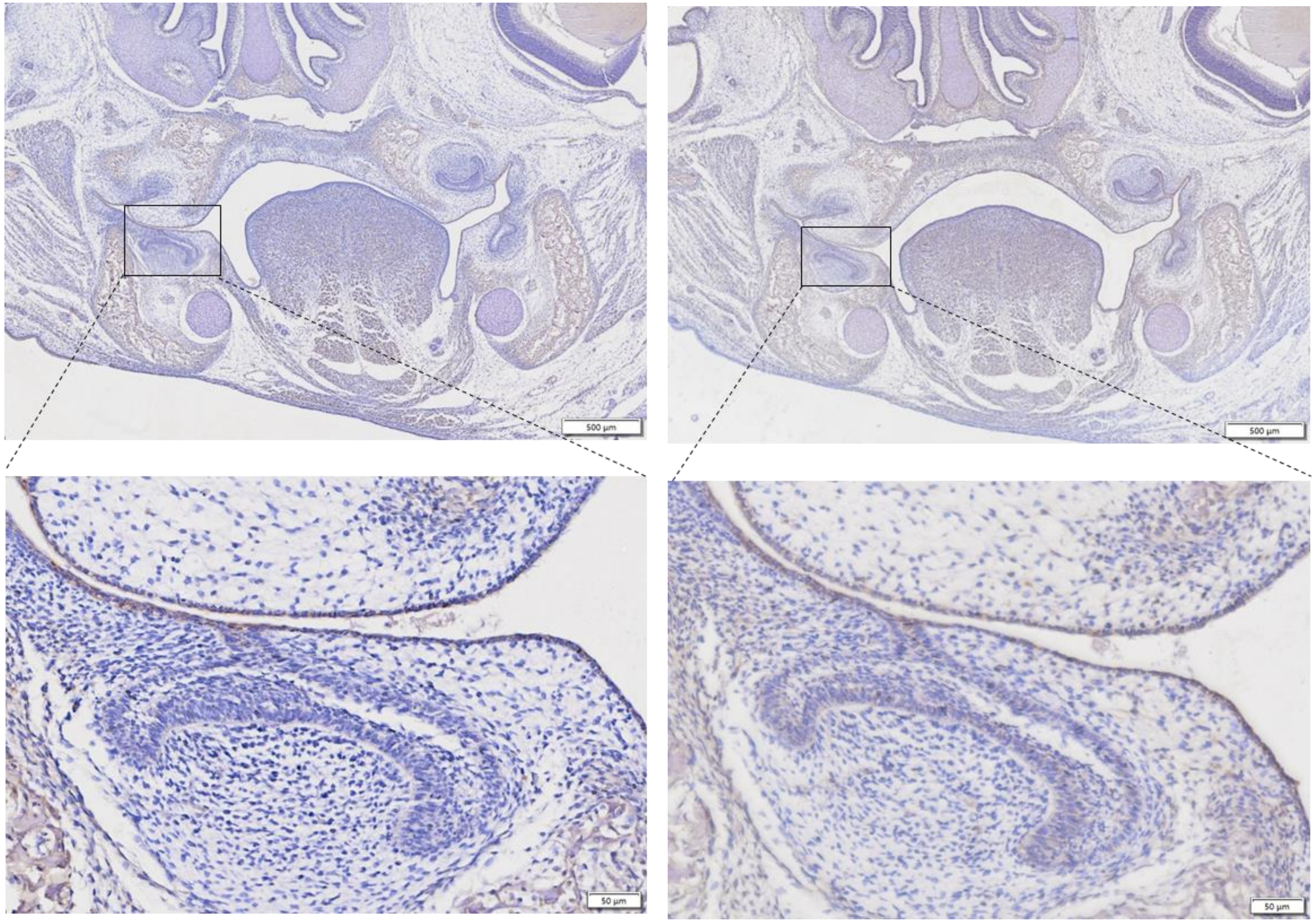

E14.5d

per1

cry1

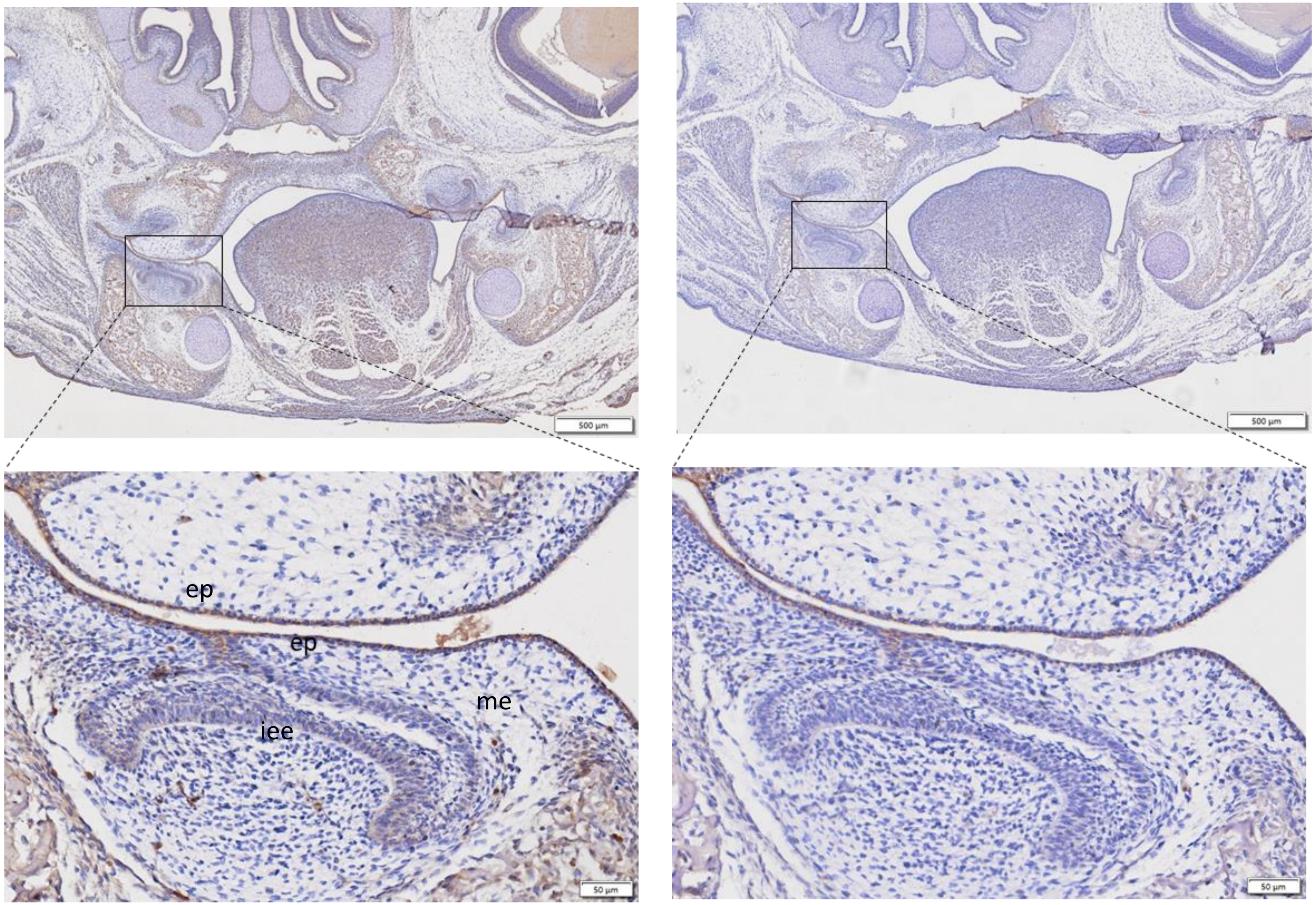

E14.5d

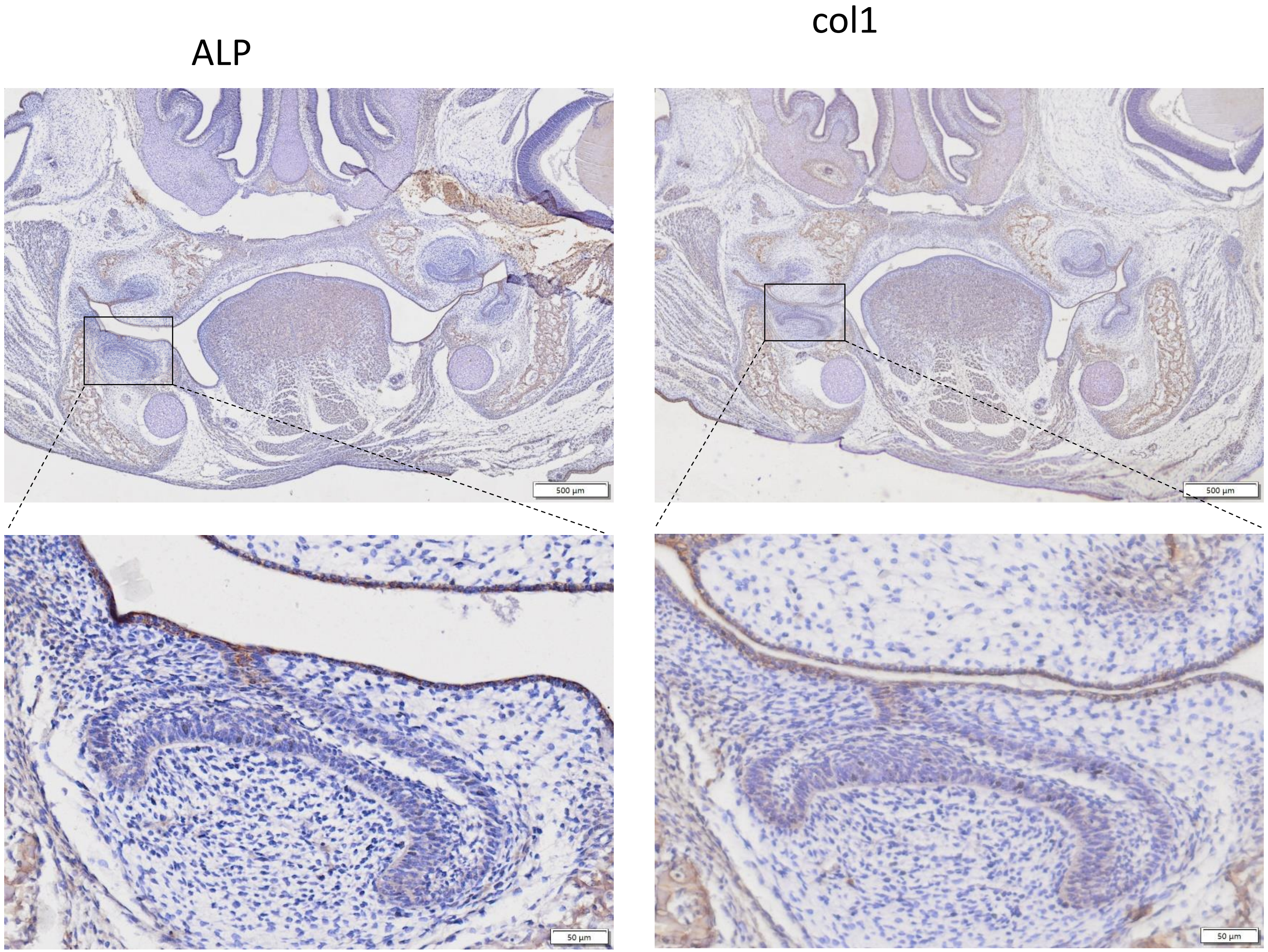

E16.5d

HE P75NTR

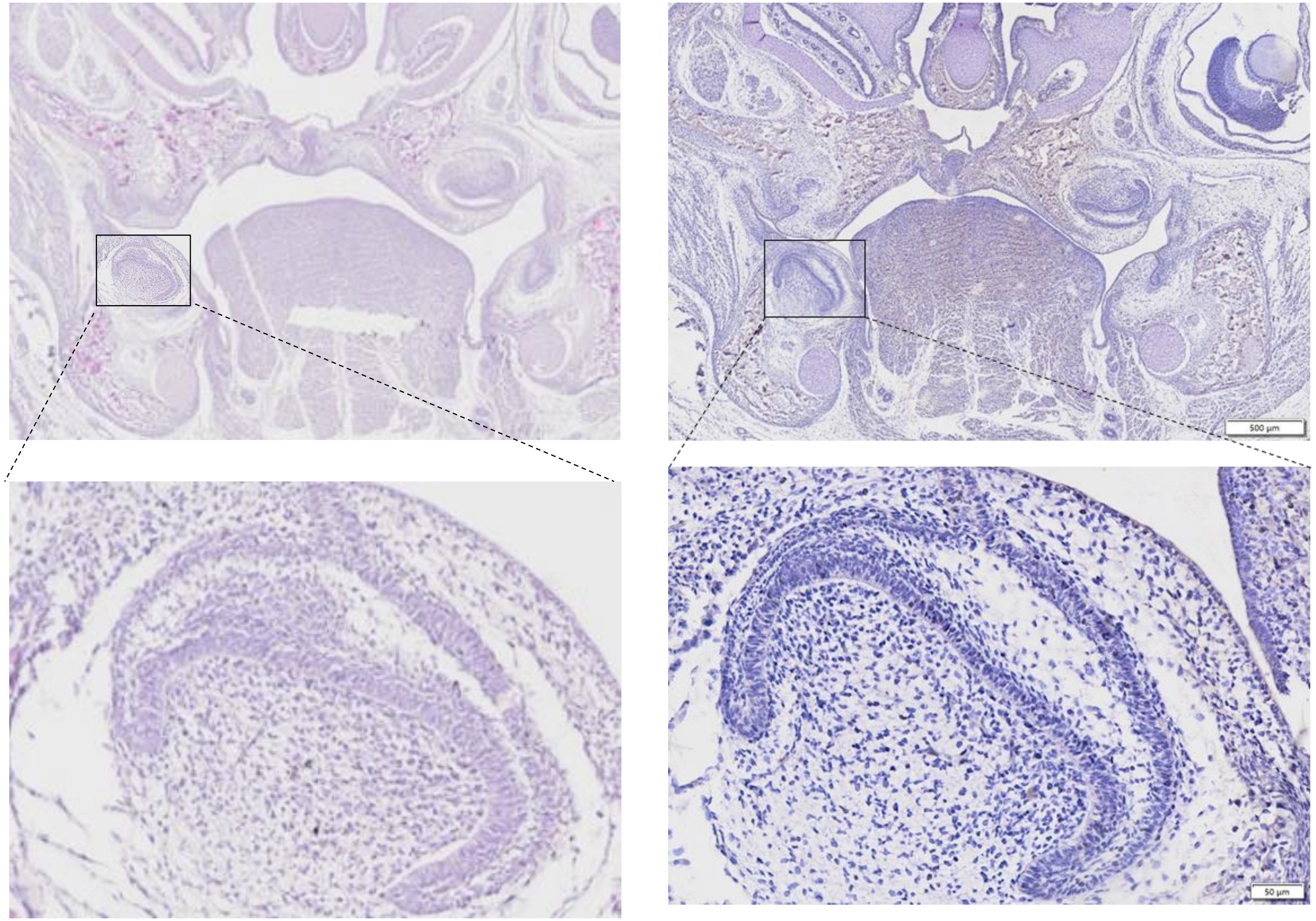

E16.5d

per1 Bmal1 clock

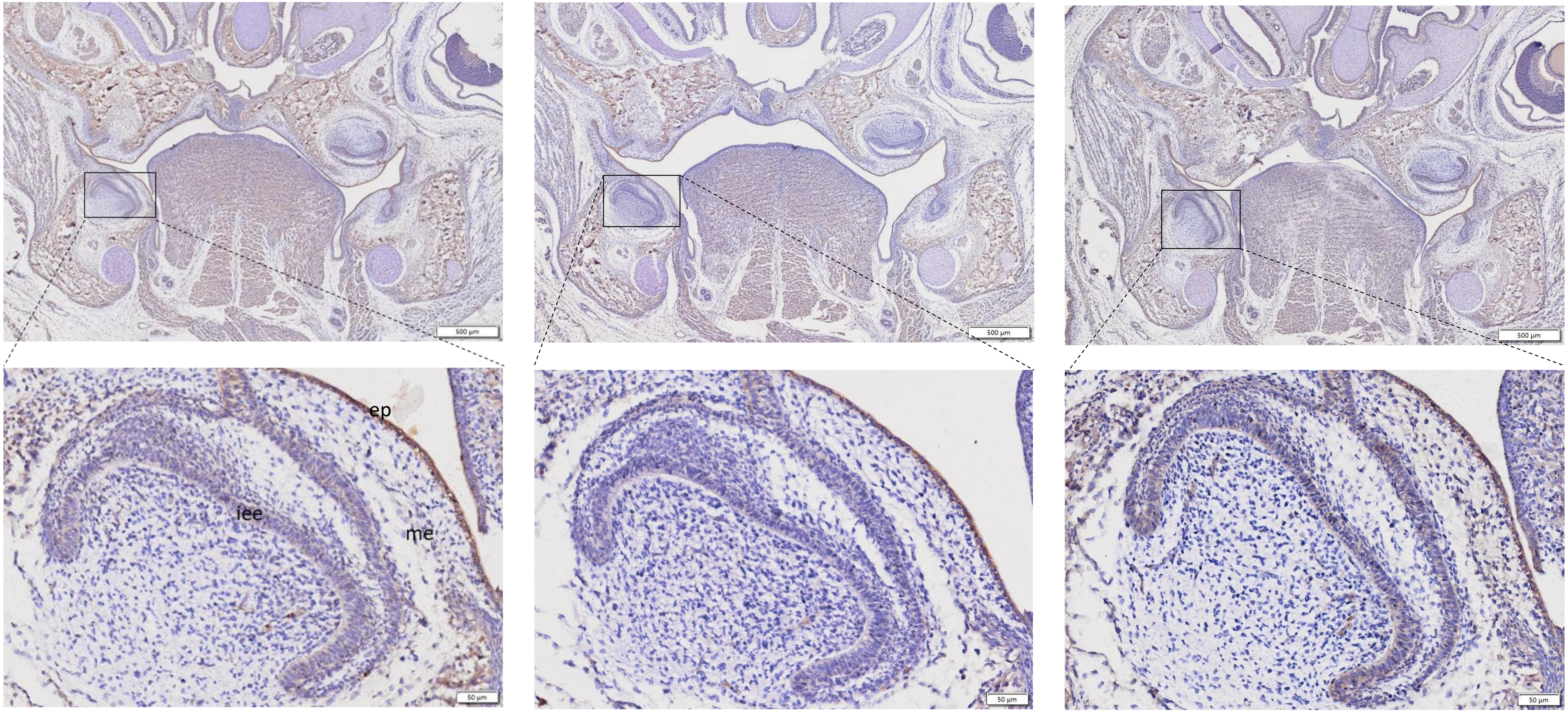

E16.5d

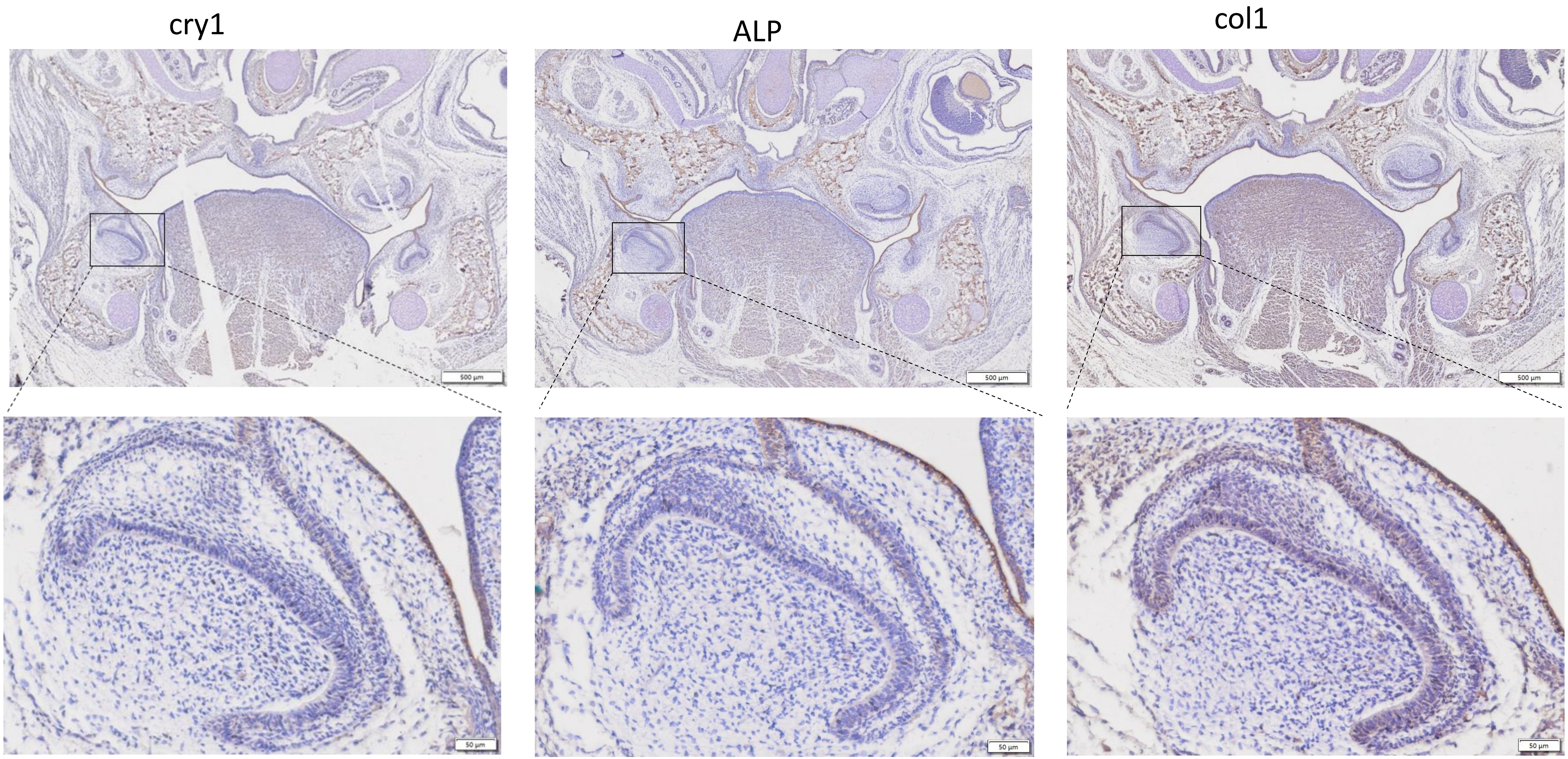

E18.5d

HE

P75NTR

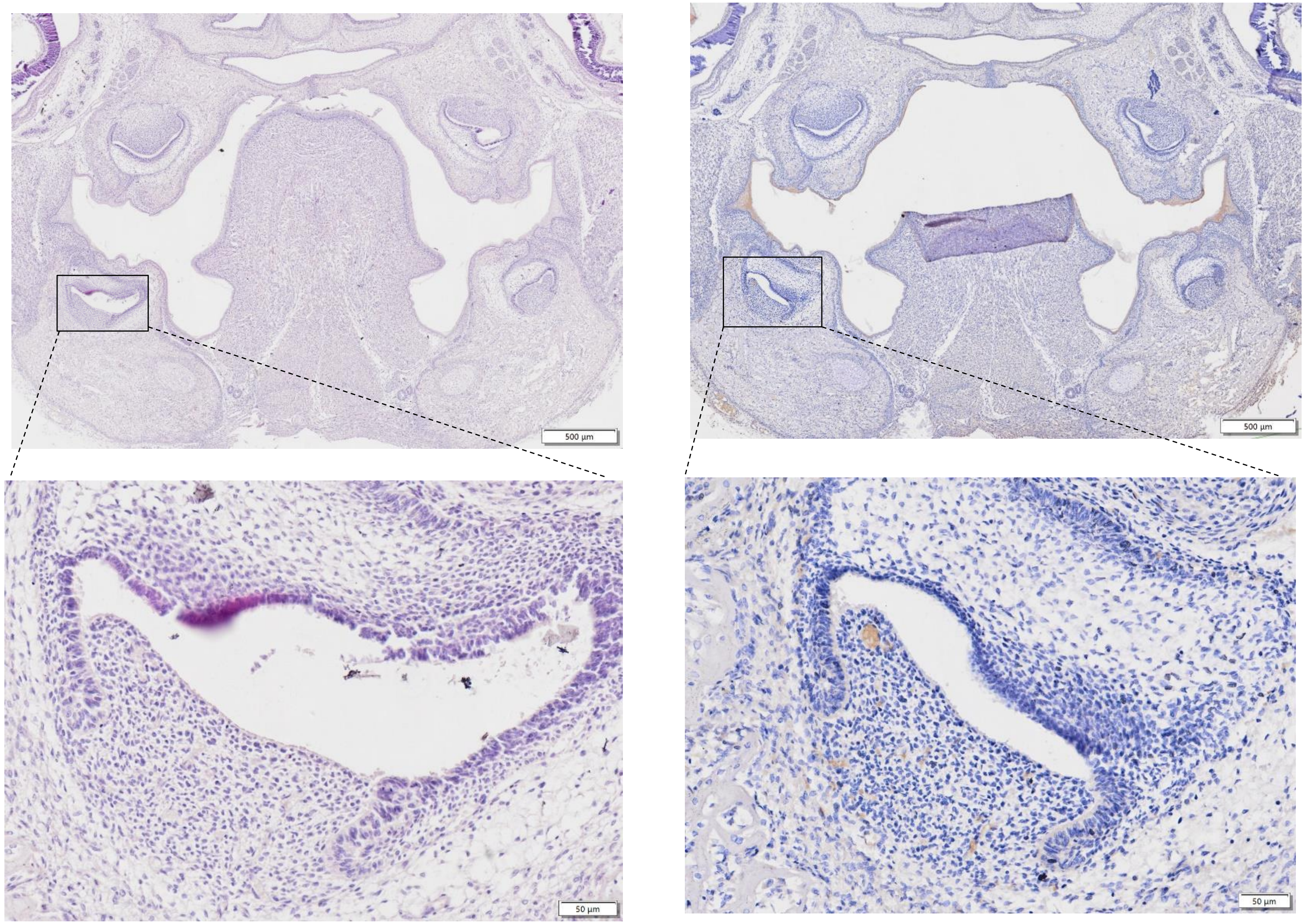

E18.5d

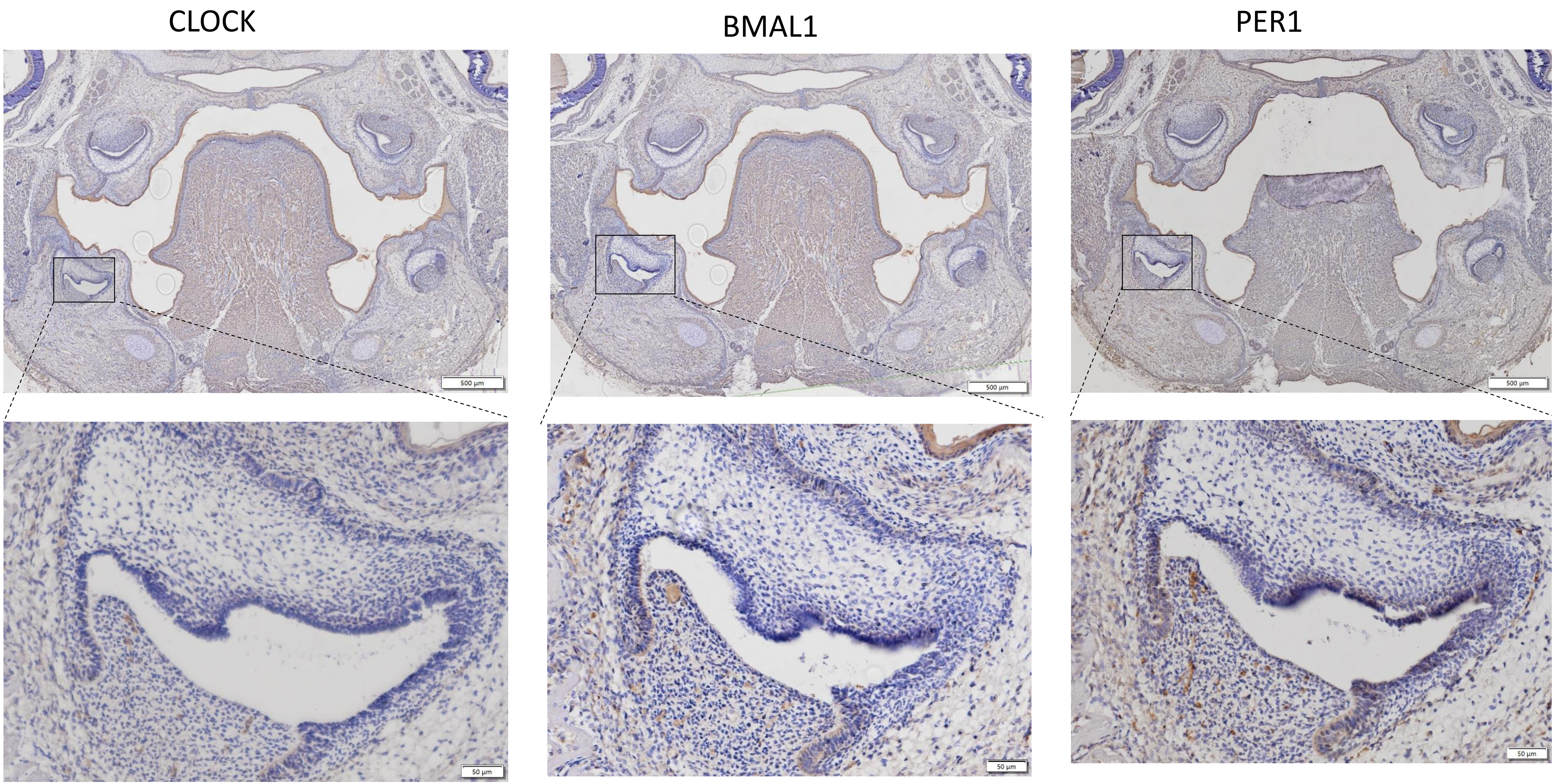

E18.5d

CRY1 ALP COL1

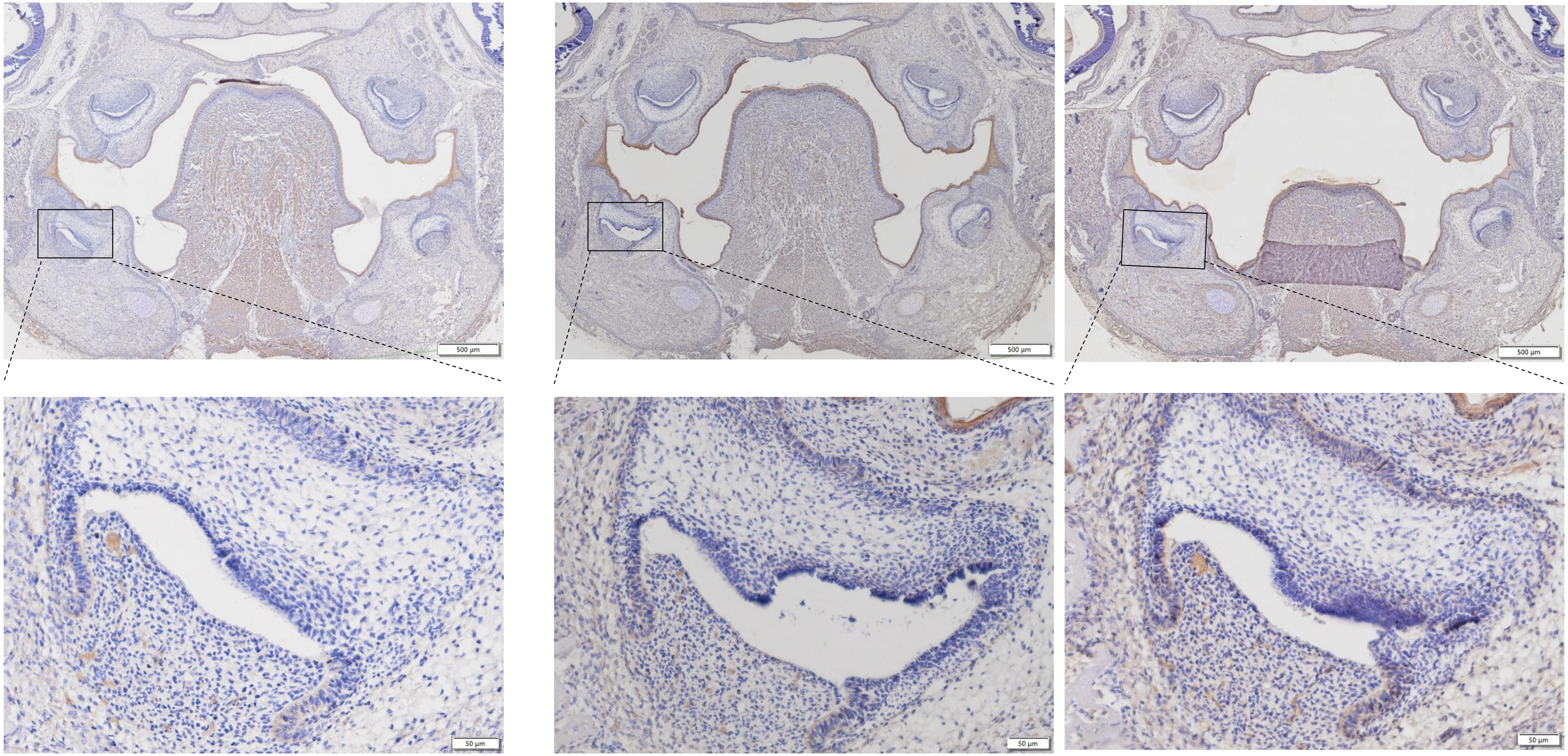

Supplement: Supplementary file 1 [file DataSheet1.ZIP › supplimengtary/FIG.1/FIG 1 supplimentary.pdf]
