# Supplementary material for: A potential role of p75NTR in the regulation of circadian rhythm and incremental growth lines during tooth development
Source: Front Physiol. 2022 Sep 23;13:981311. doi: 10.3389/fphys.2022.981311 (PMC9539461; doi:10.3389/fphys.2022.981311)

PN 4d

cry1 HE

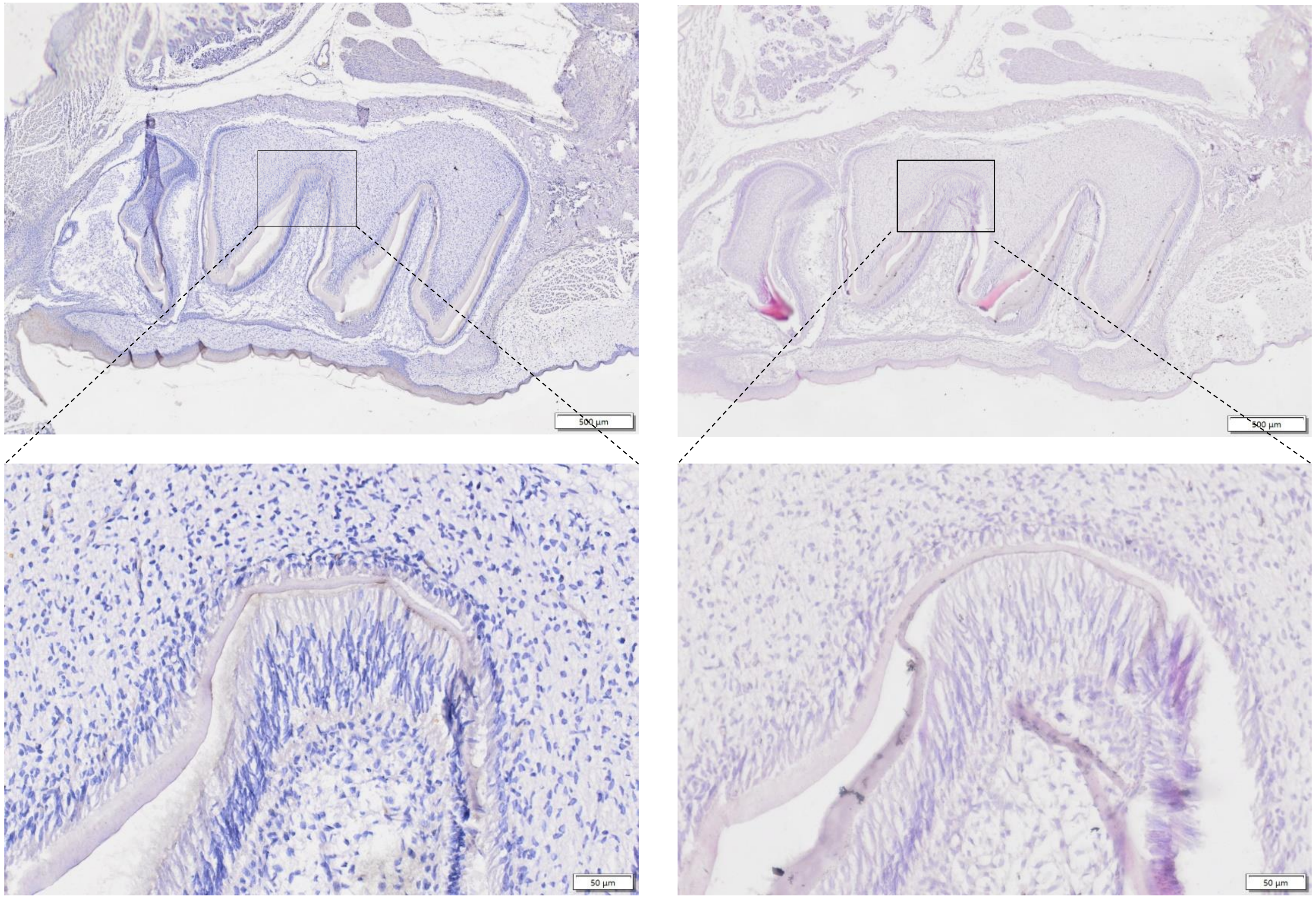

PN4d

Bmal1 clock

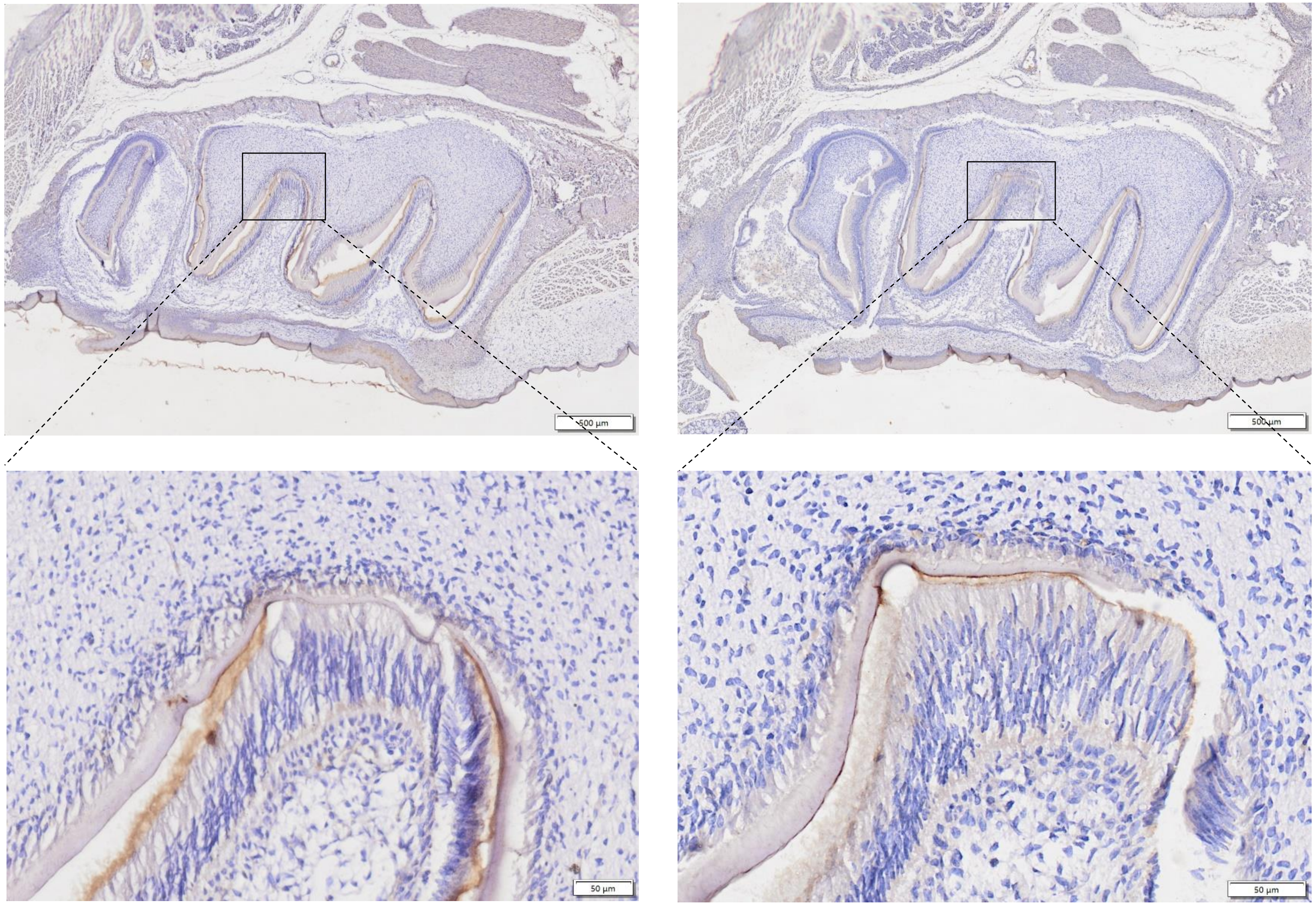

PN 4d

per1 p75NTR

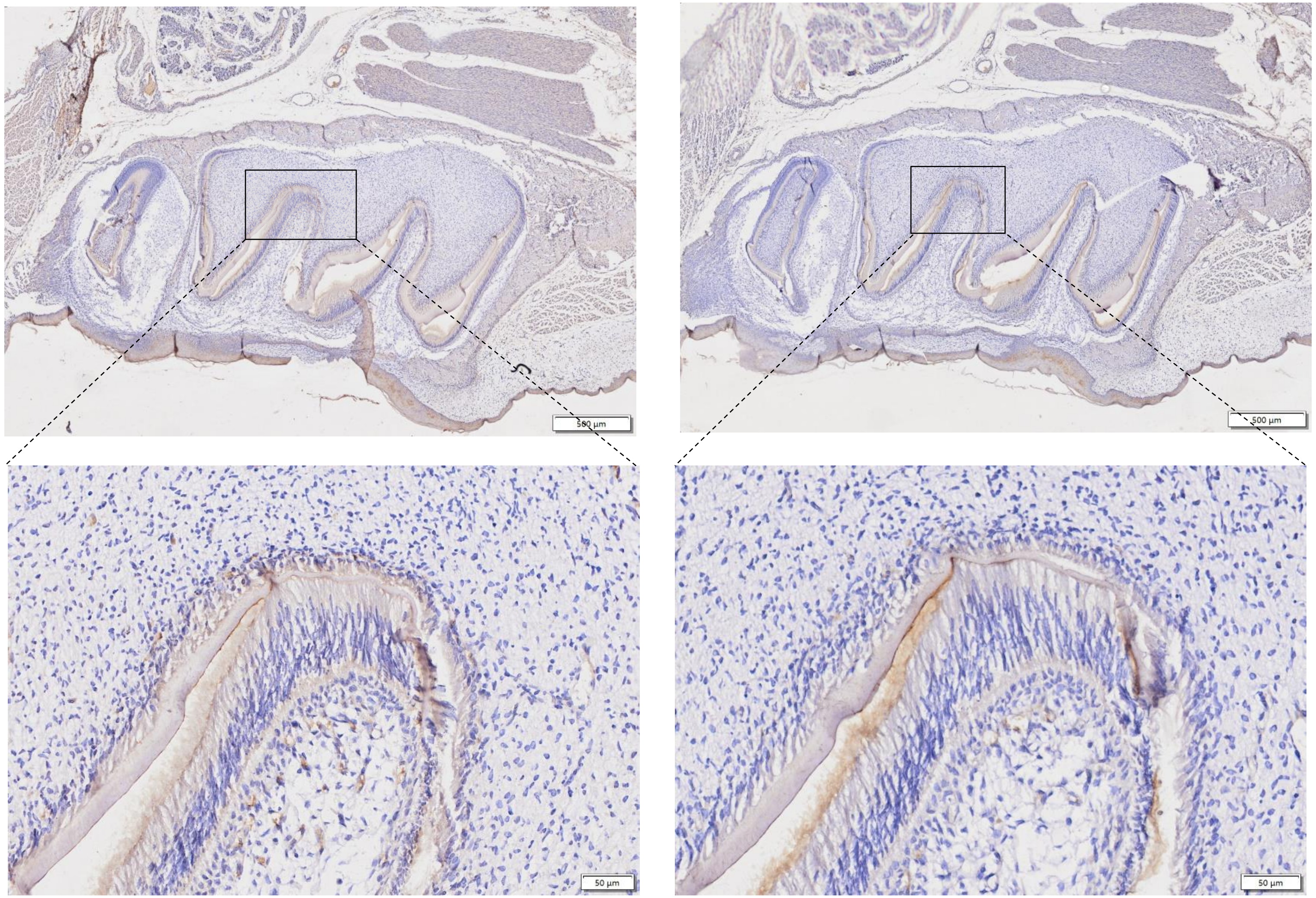

PN4d

ALP　　　　　　　　col1

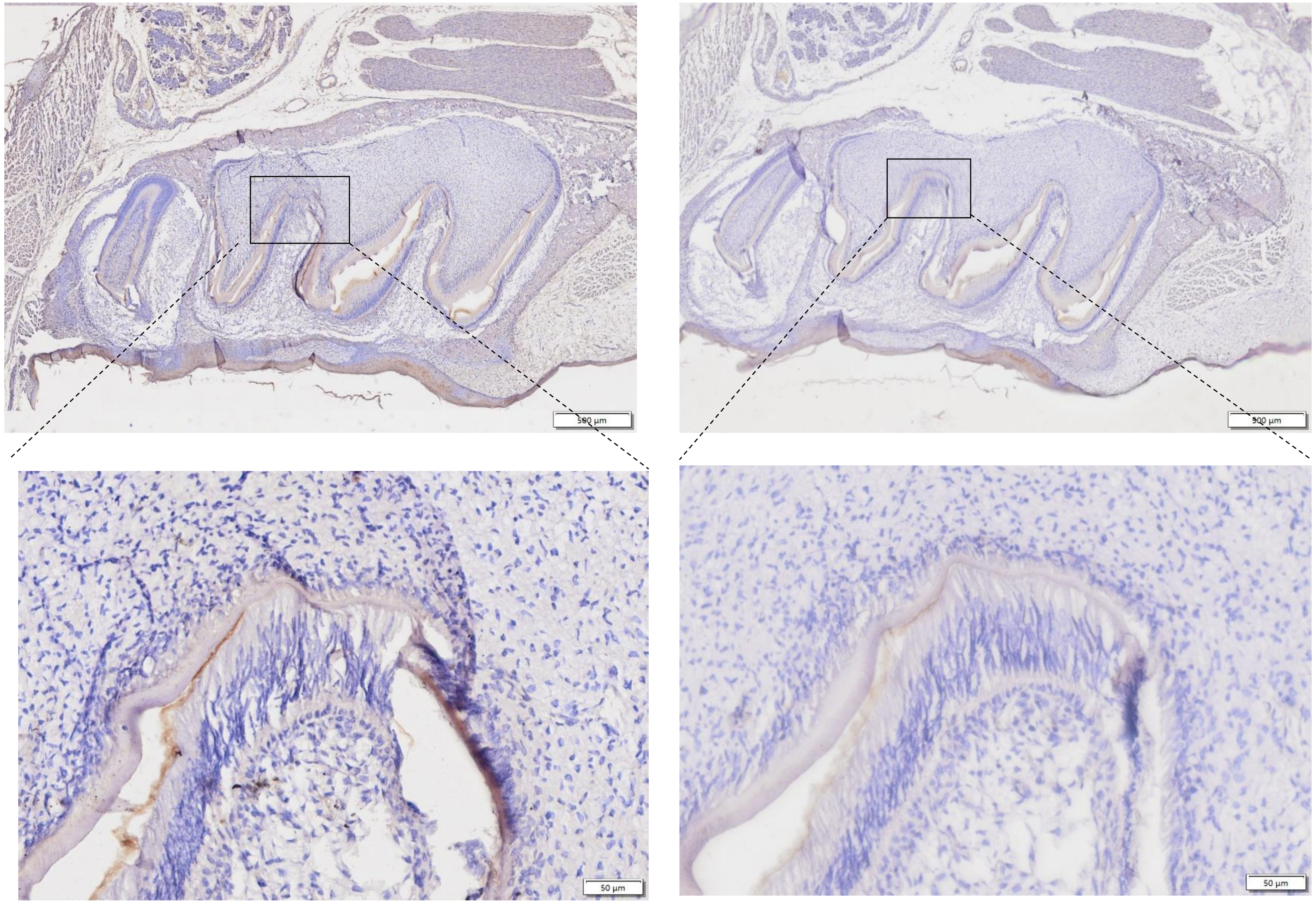

PN7d

CRY1

HE

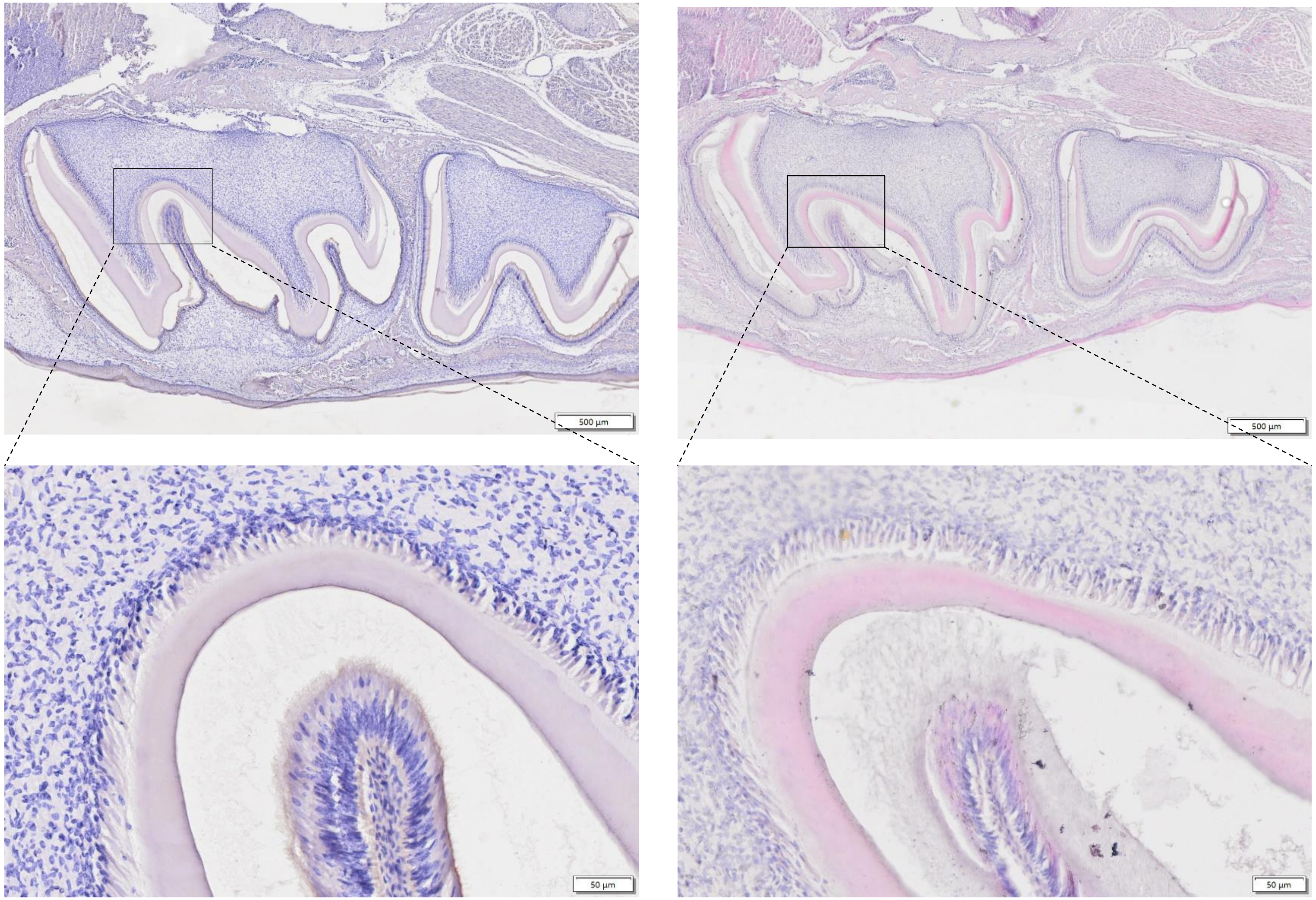

PN7d

Bmal1 | clock

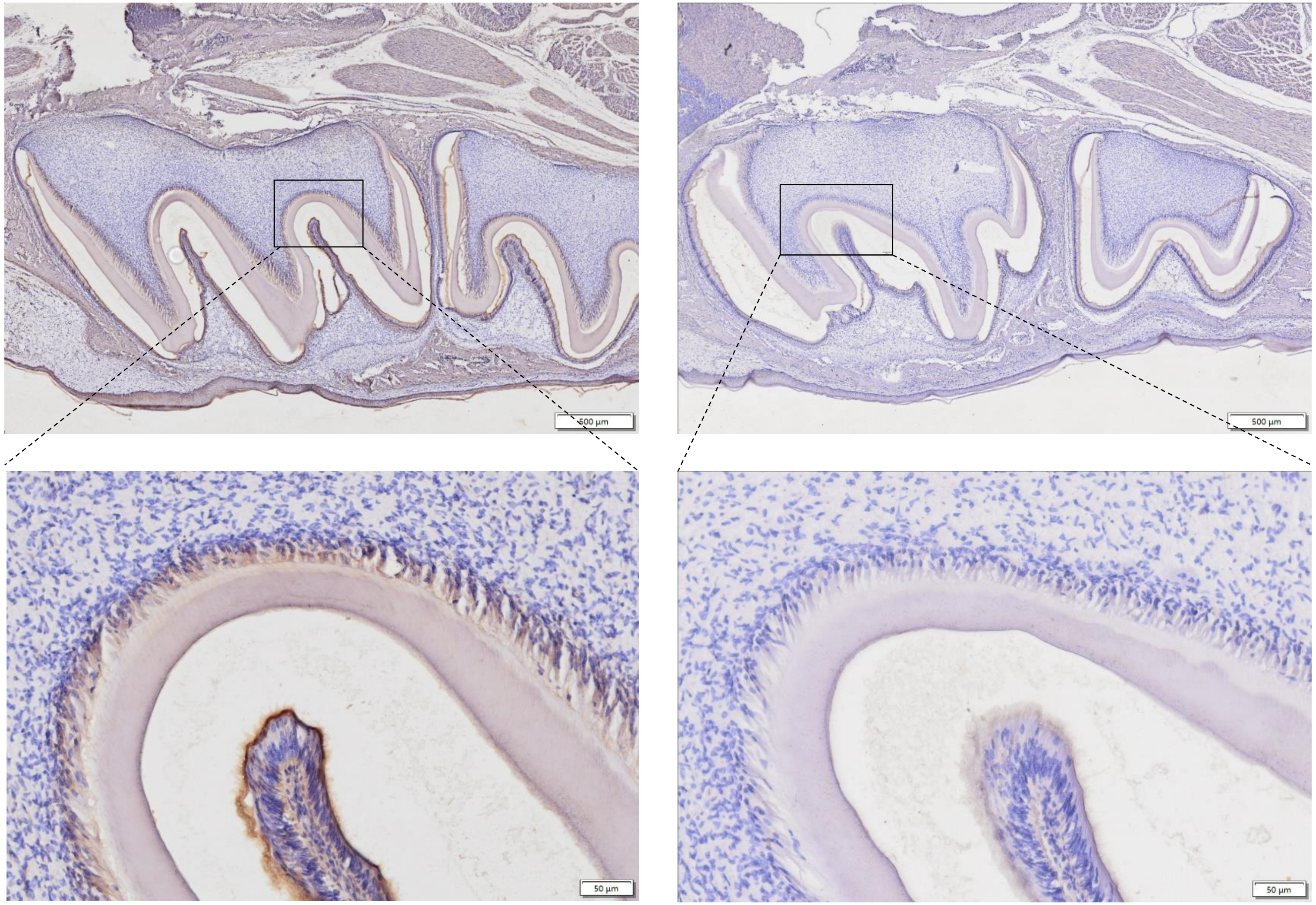

PN7d

per1 P75NTR

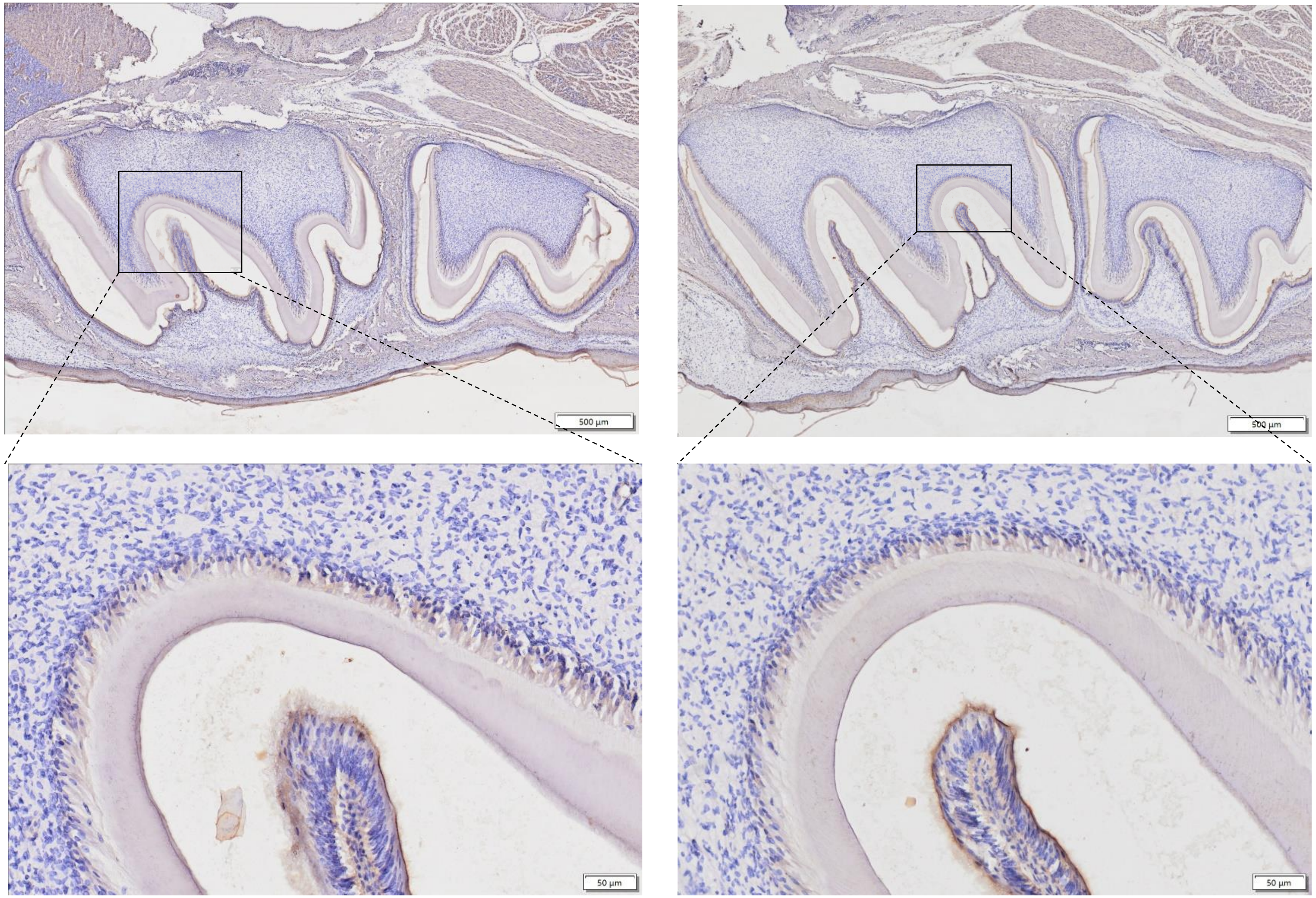

PN7d

ALP　　　　　　　　　　col1

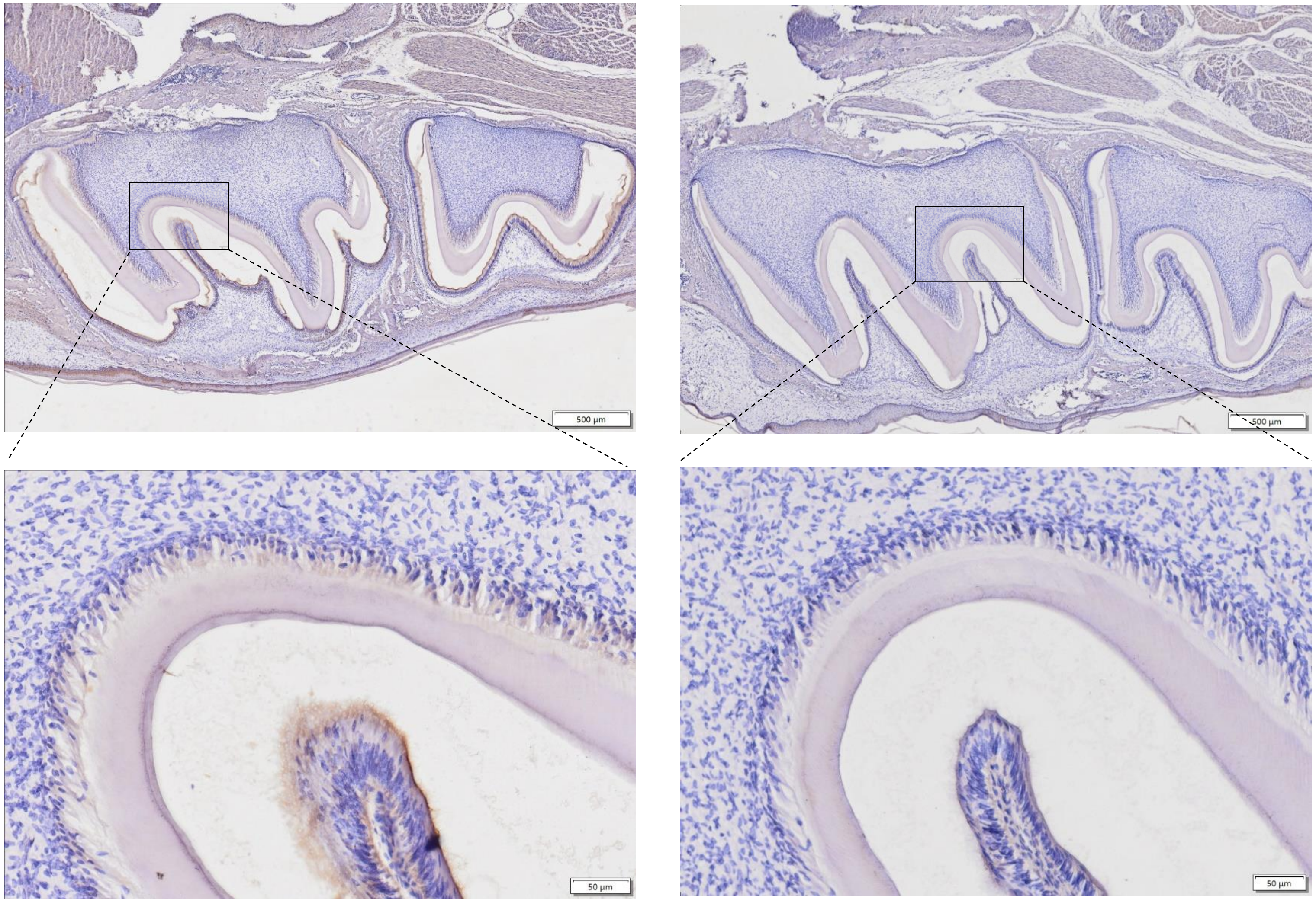

ALP表达较col1强

PN10d

cry1 HE

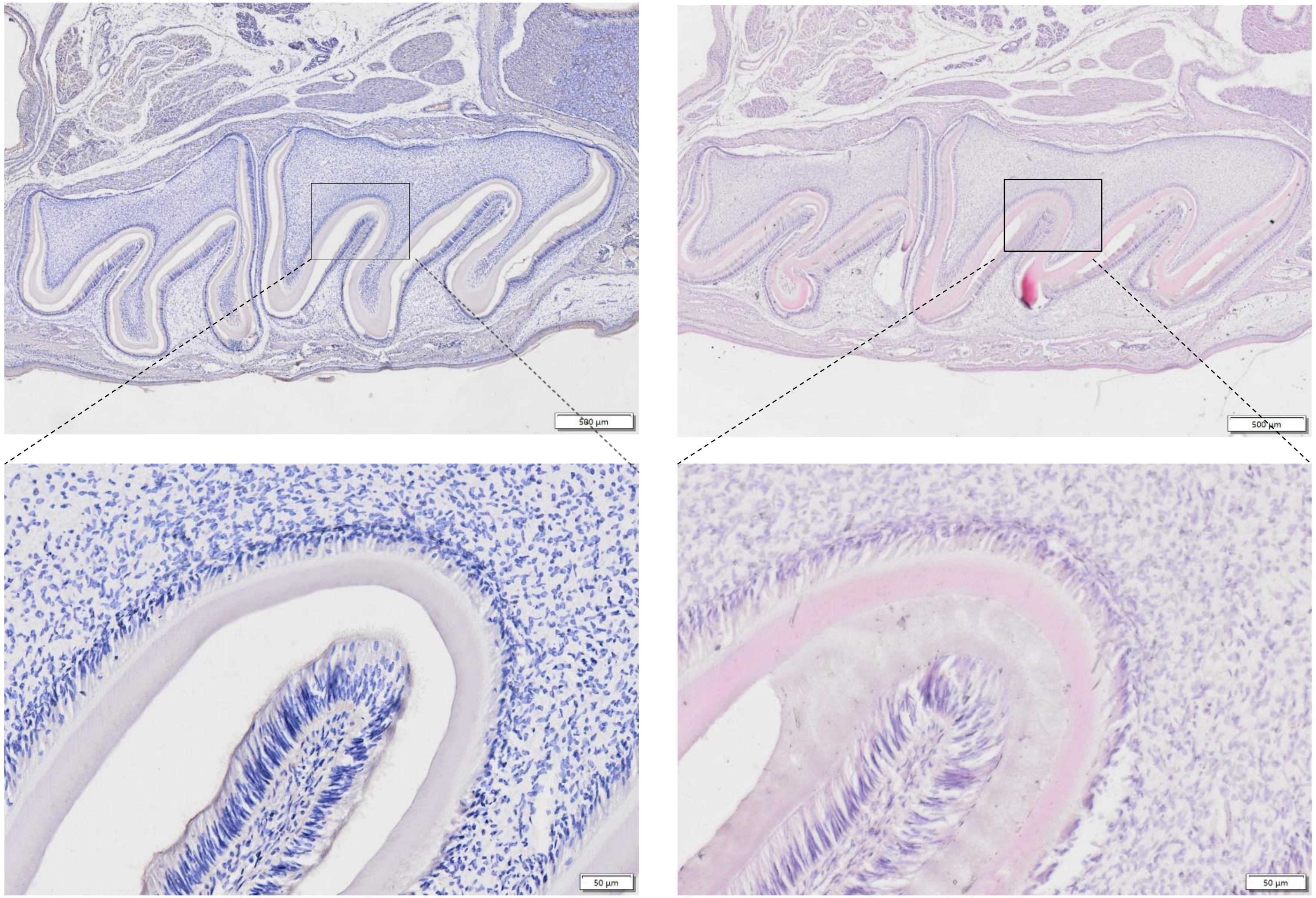

PN10d

Bmal1 clock

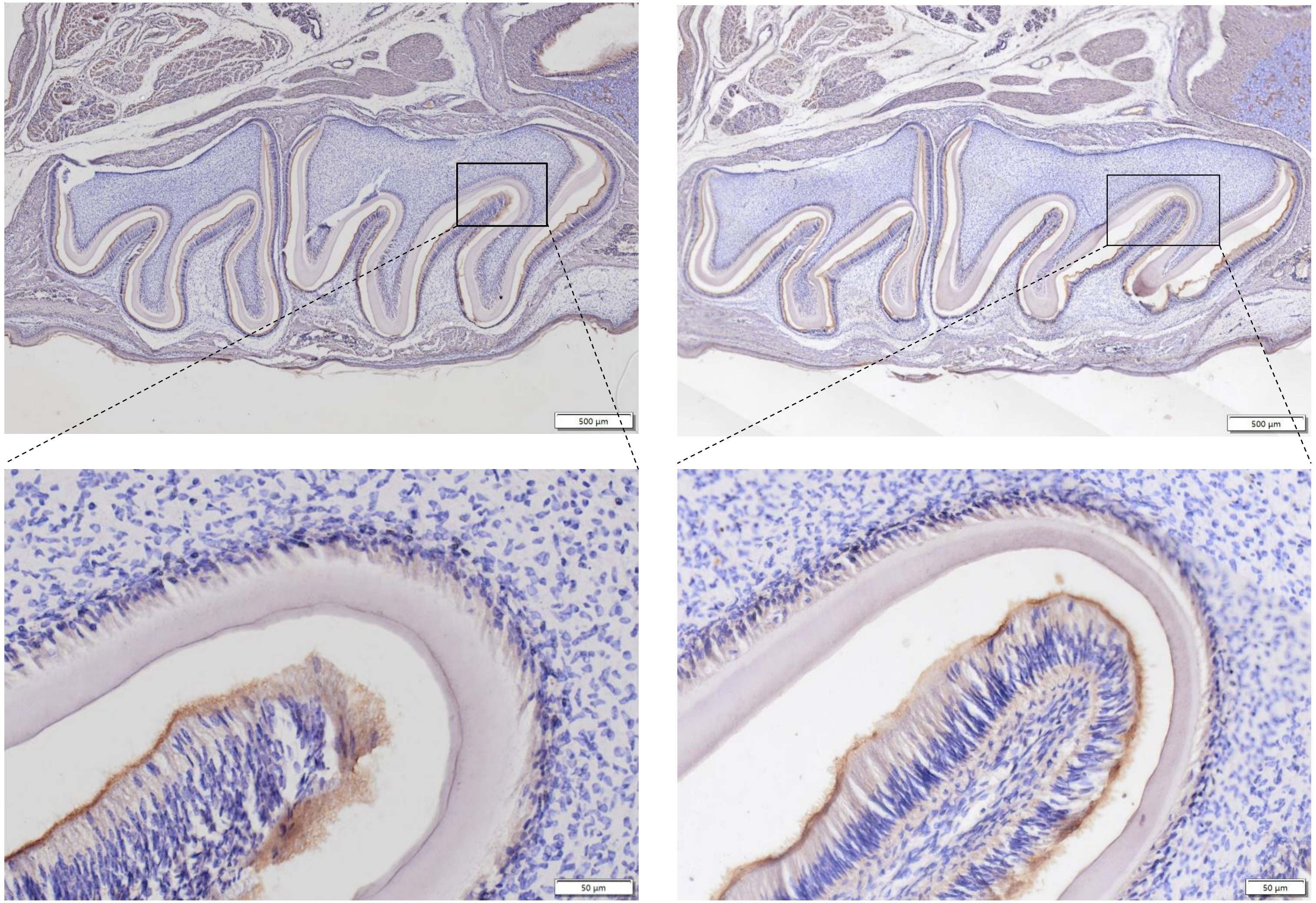

PN10d

per1 p75NTR

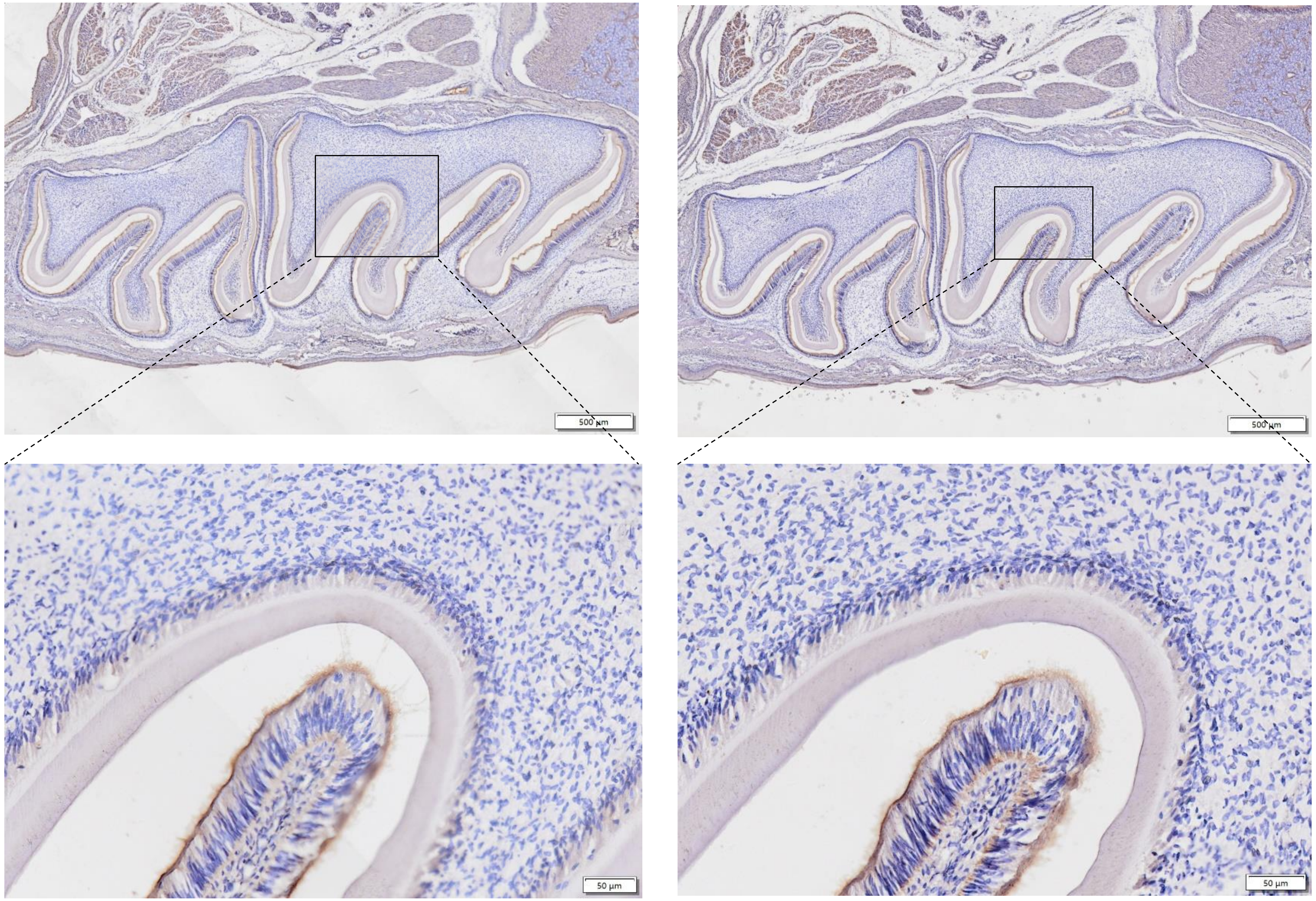

PN10d

ALP col1

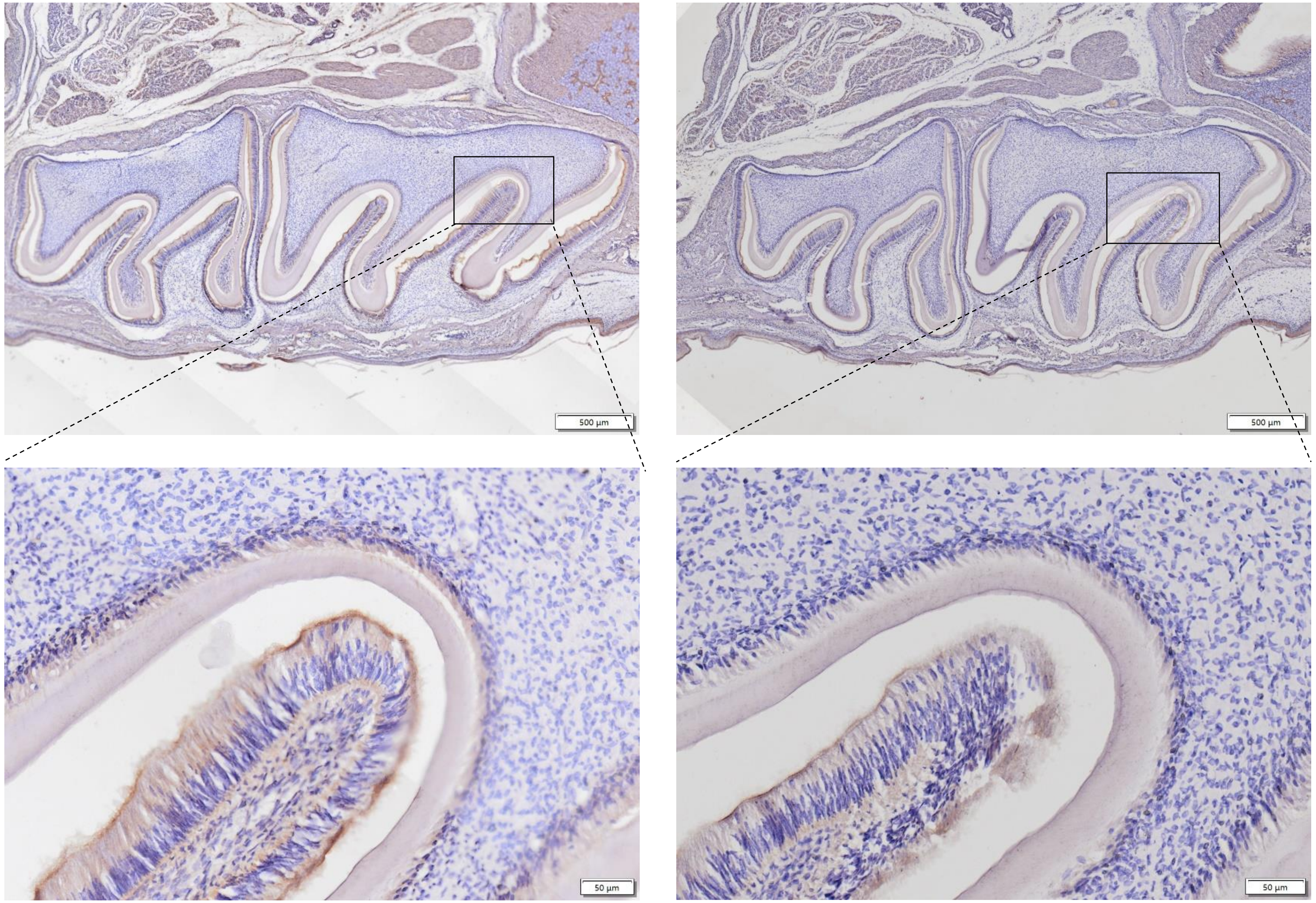

PN15d

Cry HE

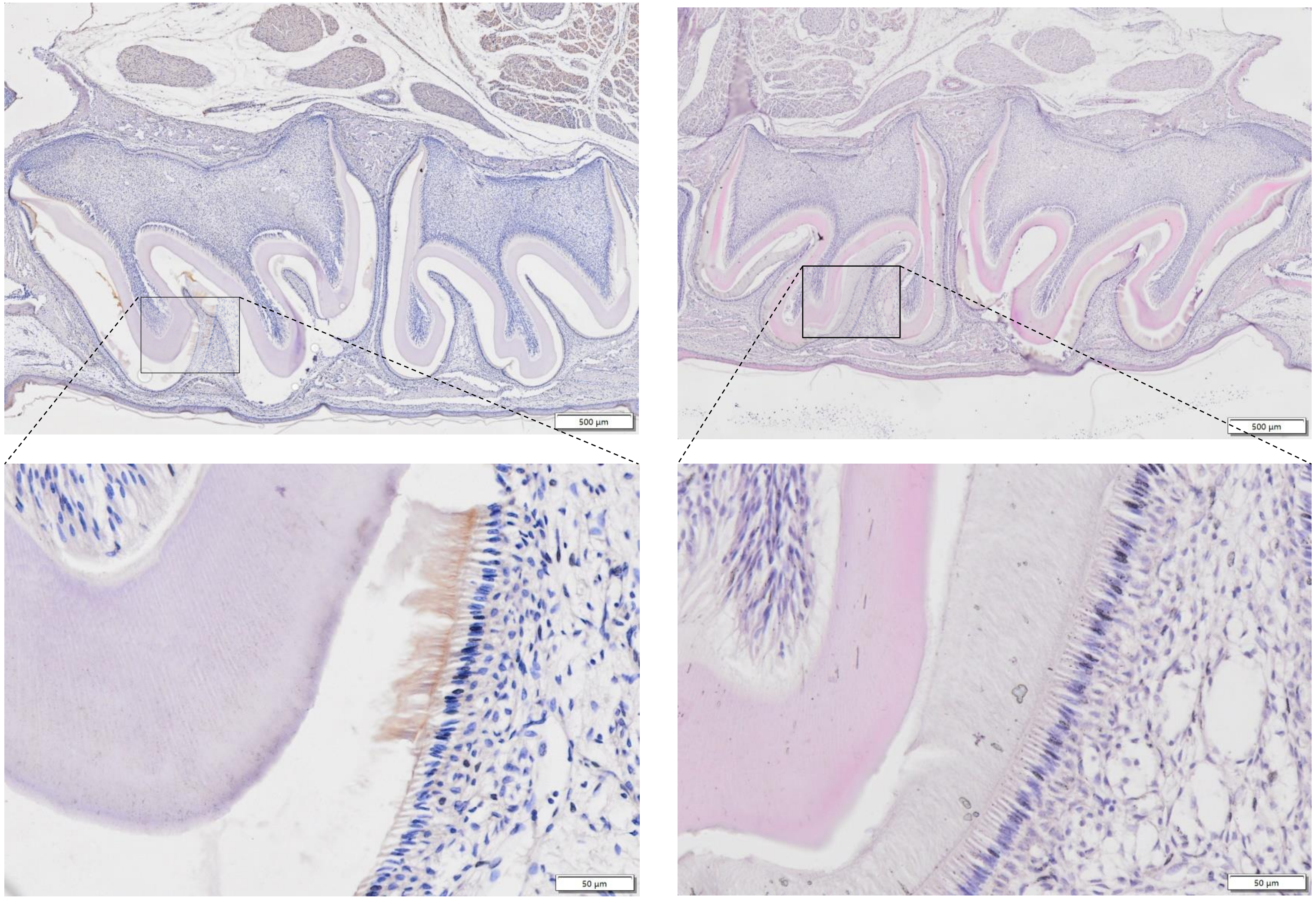

PN15d

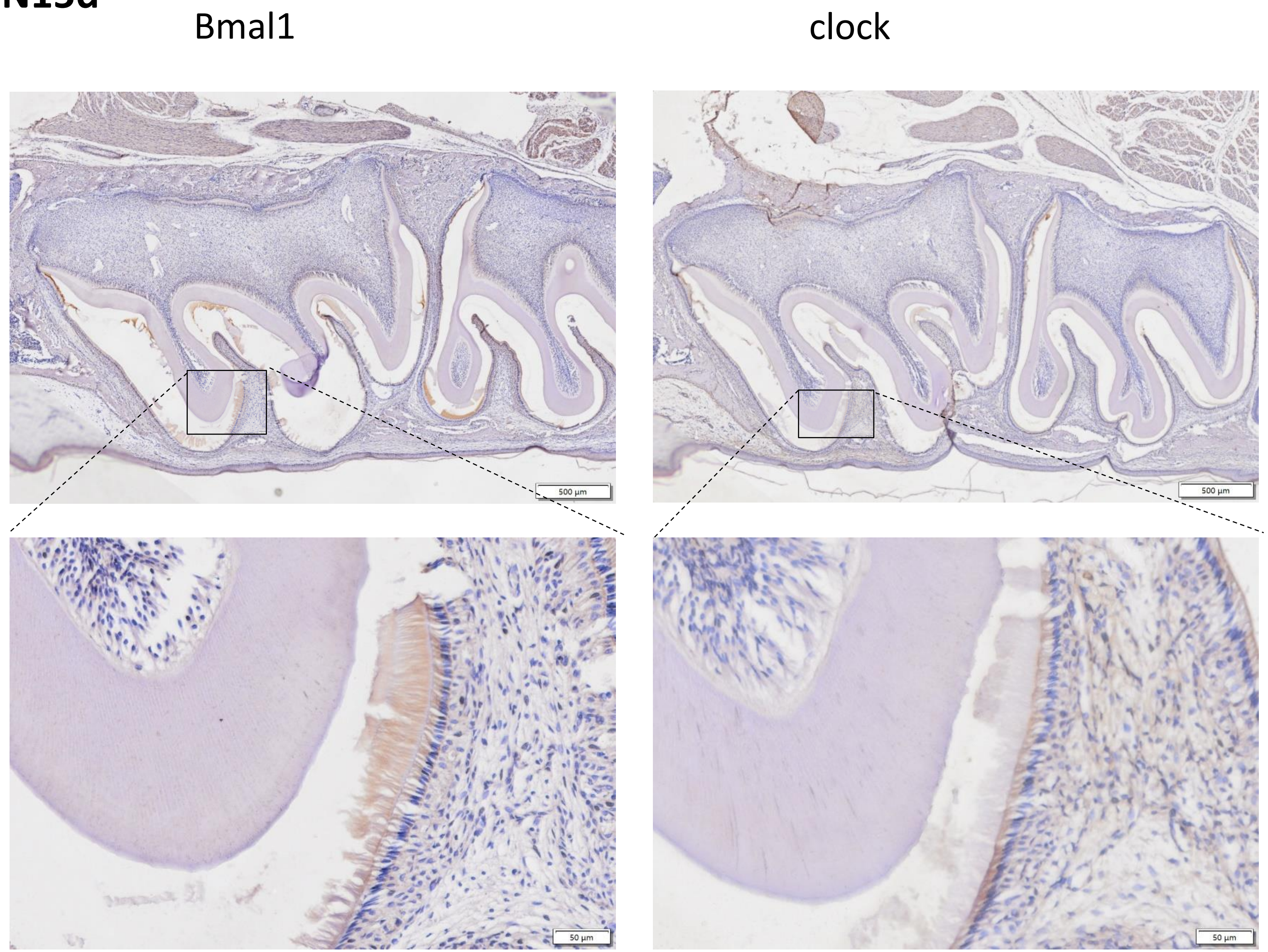

PN15d

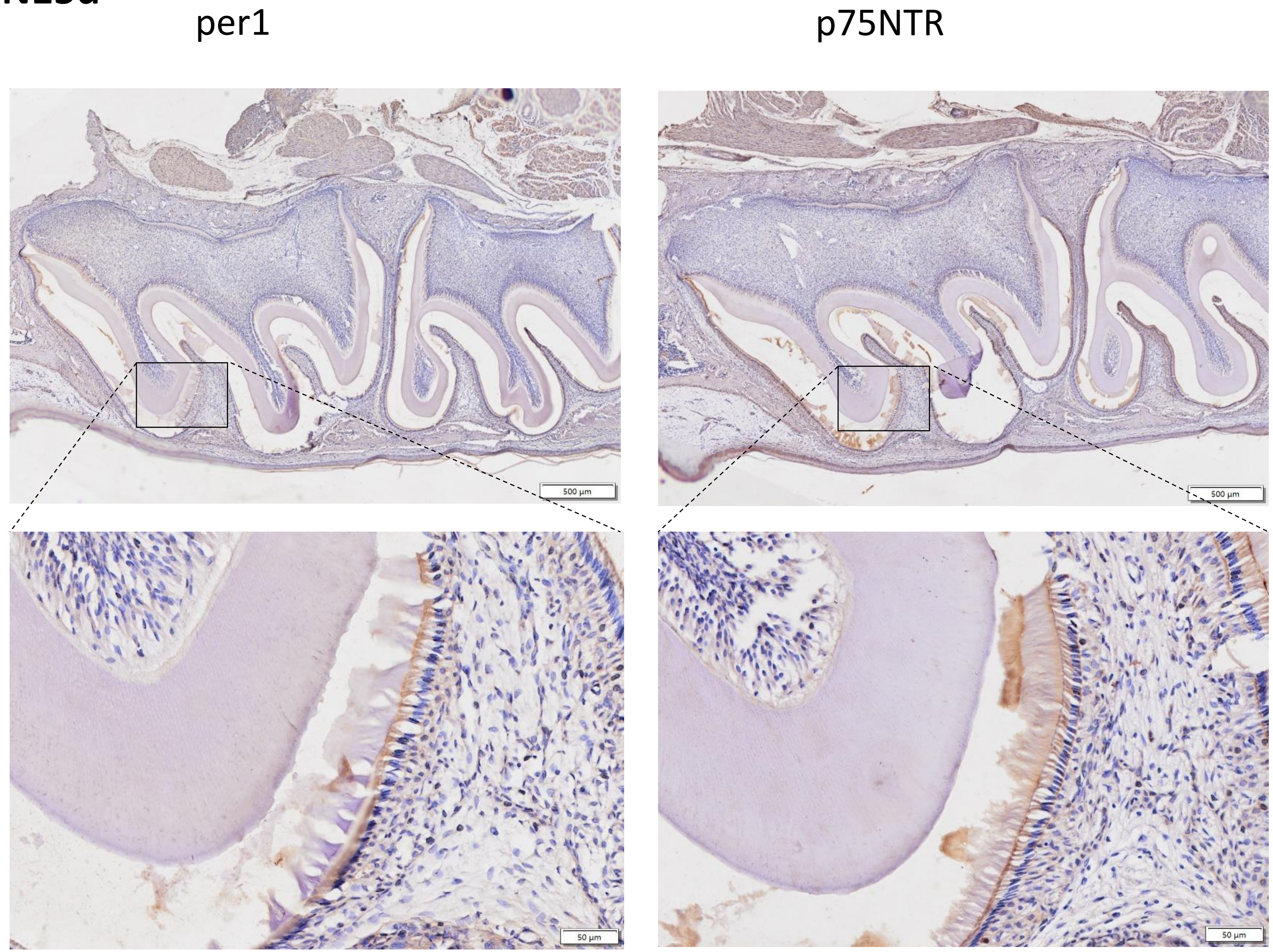

PN15d

ALP col1

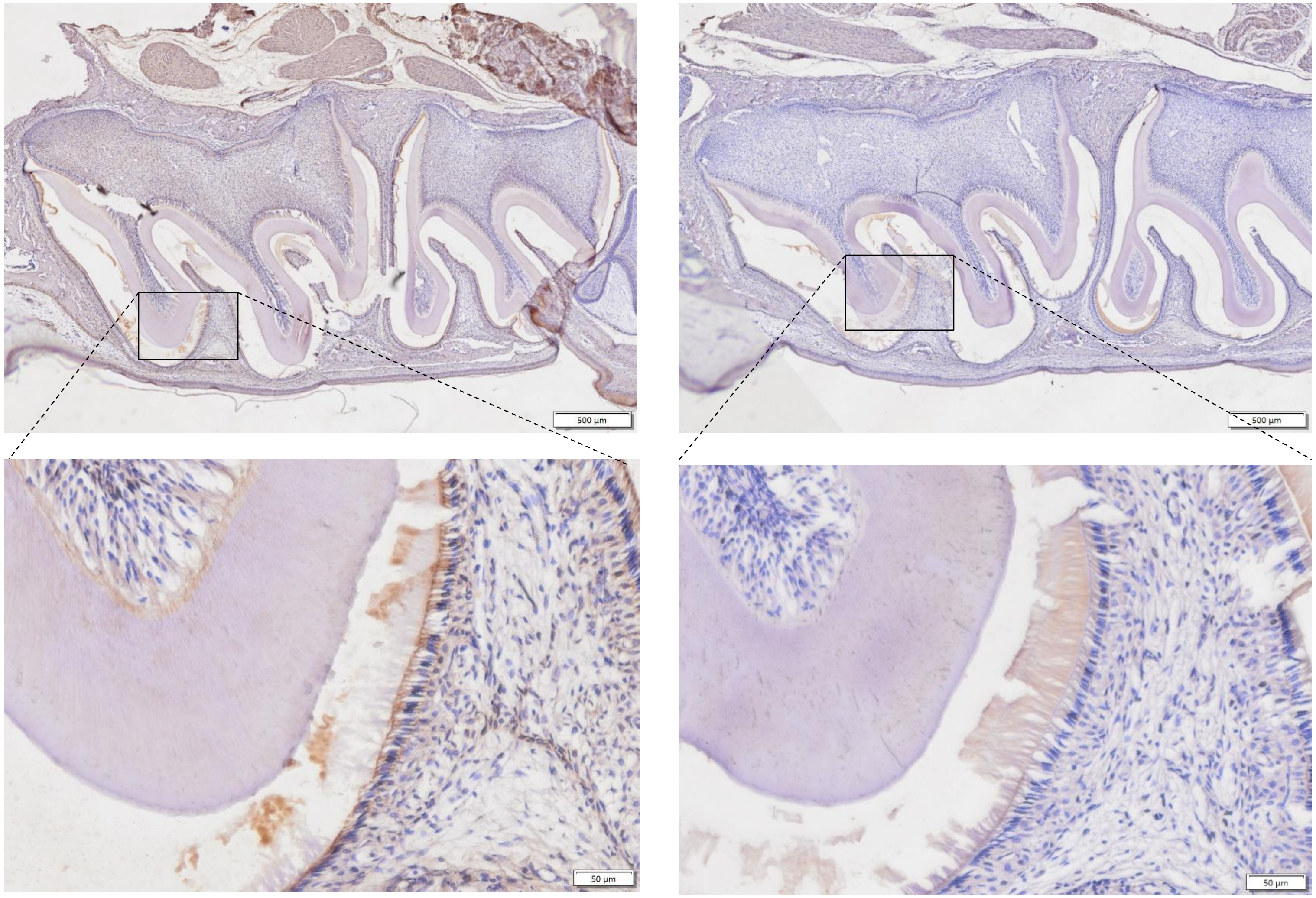

Supplement: Supplementary file 1 [file DataSheet1.ZIP › supplimengtary/FIG.2/FIG 2 supplimentary.pdf]
