# Supplementary figures and images for: A potential role of p75NTR in the regulation of circadian rhythm and incremental growth lines during tooth development
Source: Front Physiol. 2022 Sep 23;13:981311. doi: 10.3389/fphys.2022.981311 (PMC9539461; doi:10.3389/fphys.2022.981311)

**H.E.****p75NTR****Bmal1****clock****Per1****Cy1****ALP****Col1****PN4d**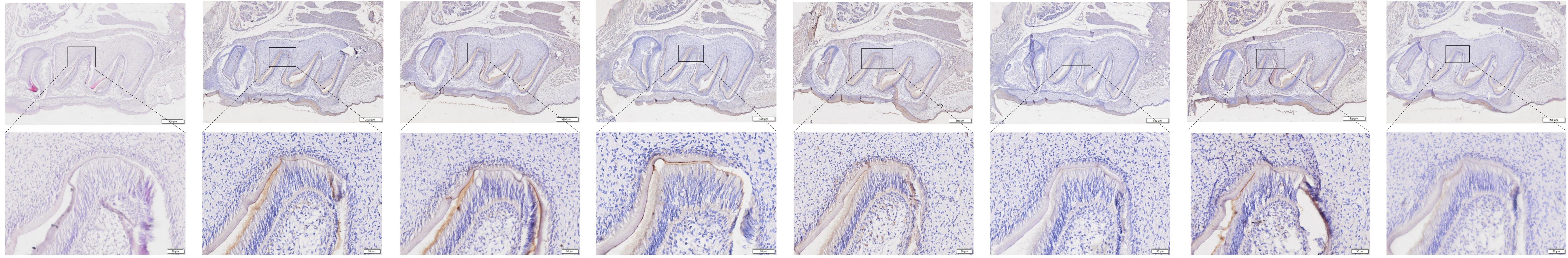**PN7d**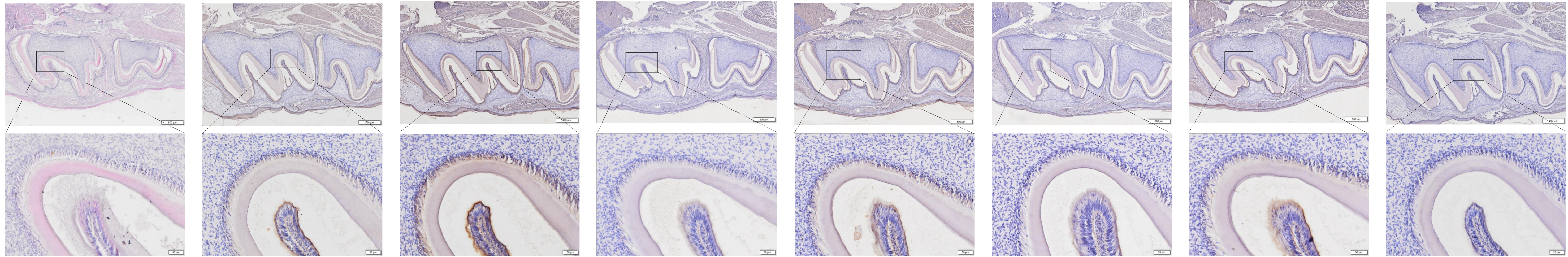**PN10d**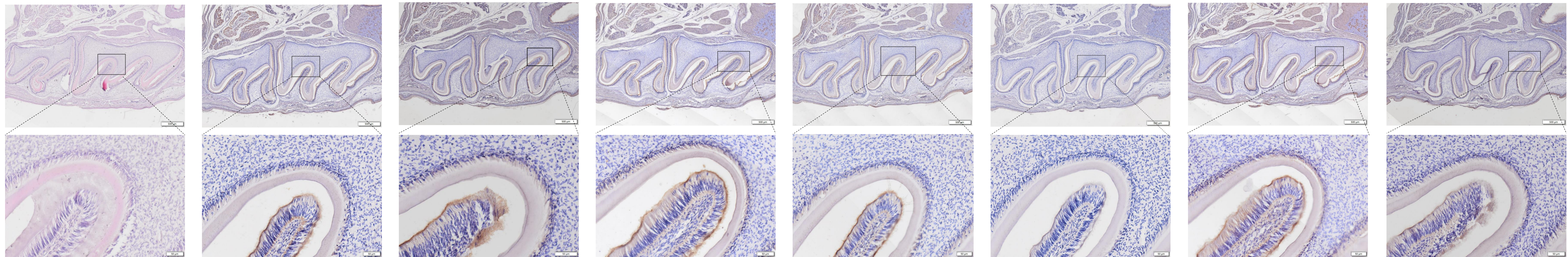**PN15d**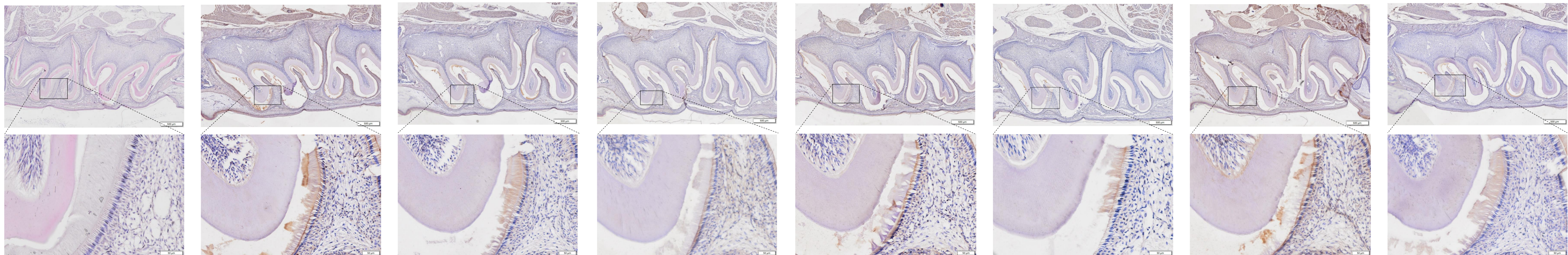

Supplement: Supplementary file 1 [file DataSheet1.ZIP › supplimengtary/FIG.2/2.pdf]

## Slide 1
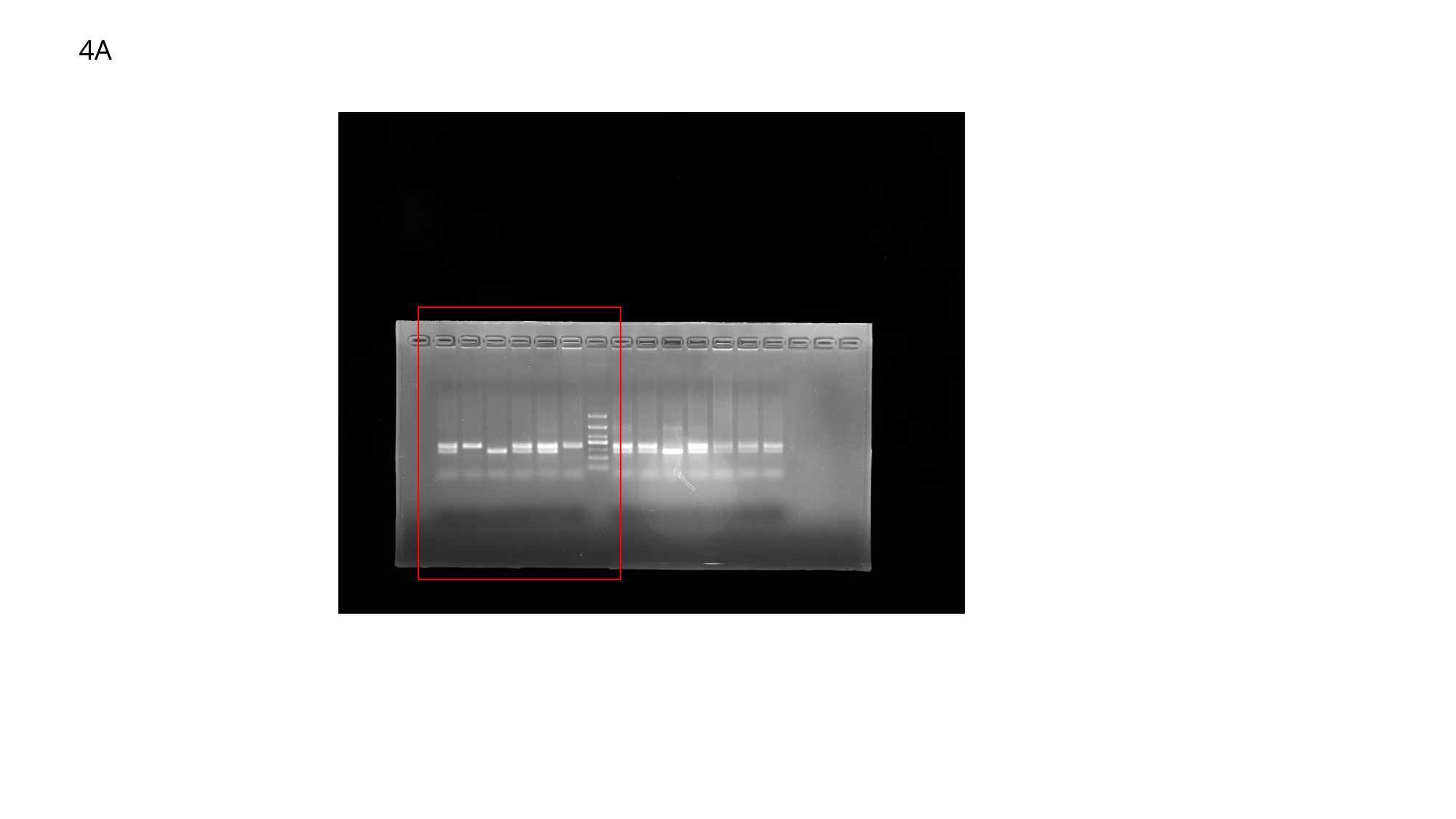

4A

## Slide 2
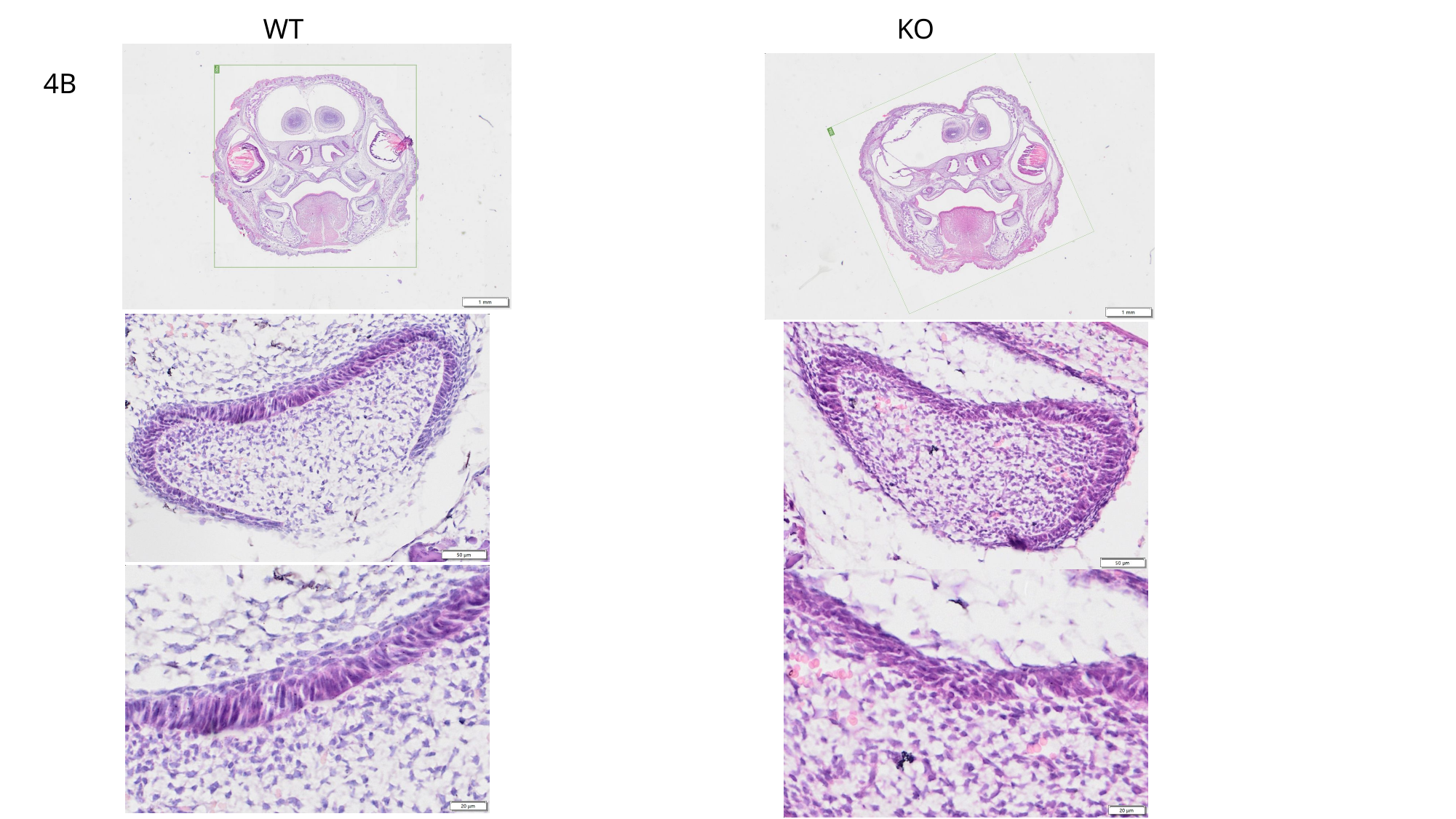

WT
KO
4B

## Slide 3
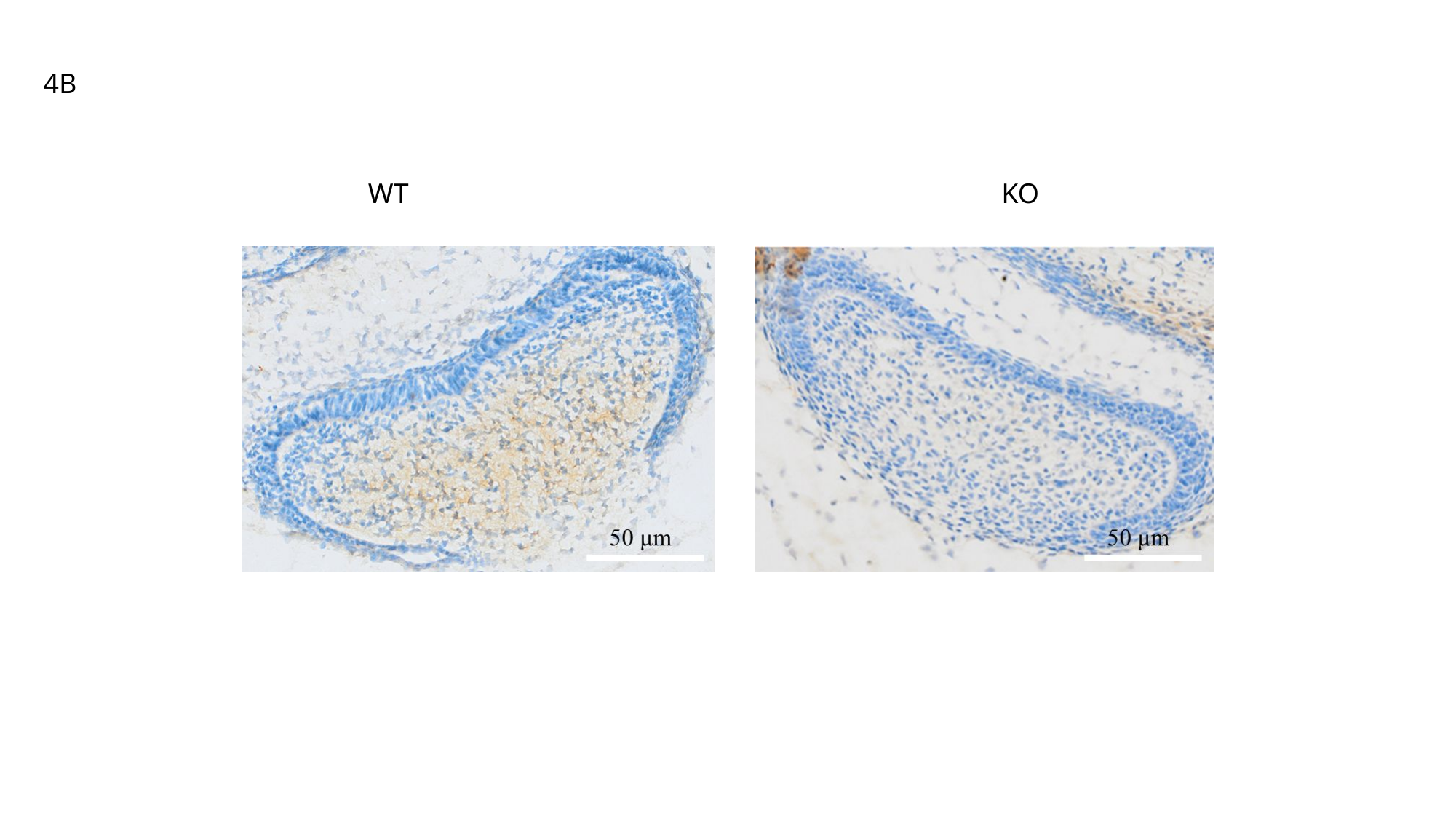

4B
WT
KO

Supplement: Supplementary file 1 [file DataSheet1.ZIP › supplimengtary/FIG.4/fig.4.pptx]
