# Supplementary material for: A potential role of p75NTR in the regulation of circadian rhythm and incremental growth lines during tooth development
Source: Front Physiol. 2022 Sep 23;13:981311. doi: 10.3389/fphys.2022.981311 (PMC9539461; doi:10.3389/fphys.2022.981311)
Supplement: Supplementary file 1 [file DataSheet1.ZIP › supplimengtary/table.doc]

Mice

| Genes | Upstream (5’–3’) | Downstream (5’–3’) | Product Size(bp) |
| --- | --- | --- | --- |
| *GAPDH* | TGACCTCAACTACATGGTCTACA | CTTCCCATTCTCGGCCTTG | 85 |
| *p75NTR* | CCAGAGCGAGACCTCATAGC | AGATGGAGCAATAGACAGGAATG | 121 |
| *Mage-D1* | ACTTACGACCCTCACCTAATCTG | ATGGGCACCTTCGTGTAGTC | 154 |
| *Bmal1* | AAGACAATGAGCCAGACAACG | TCCCATCTATTGCGTGTCG | 147 |
| *Clock* | CCATCCAGTATGCCACAGAAC | TCACCACCTGACCCATAAGC | 172 |
| *Per1* | CCAGTACAACCAAGCGTAAATG | TTGCTGACGACGGATCTTTC | 123 |
| *Per2* | AGCGGCTTAGATTCTTTCACTC | TCTCATTCTCGTGGTGTTTCC | 88 |
| *Runx2* | TTCCAGACCAGCAGCACTCC | GCTTCCGTCAGCGTCAACAC | 189 |
| *Col1* | TAAGGGTCCCCAATGGTGAGA | GGGTCCCTCGACTCCTACAT | 170 |
| *ALP* | TGACTACCACTCGGGTGAACC | TCTGGTGGCATCTCGTTATCC | 94 |
| *Msx1* | TCCTCAAGCTGCCAGAAGATG | CTTGCGGTTGGTCTTGTGC | 155 |
| *Dlx1* | ATGCCAGAAAGTCTCAACAGC | GAAGGAGACATTTGCTGGTTG | 83 |
| *DMP1* | CAGAGGGACAGGCAAATAGTG | CATCGCCAAAGGTATCATCTC | 168 |
| *Dspp* | GGACACAGCAGGATAGGTAGCAG | CACTTTCGTCACTTCCGTTAGATTC | 124 |

rat

| Genes | Upstream (5’–3’) | Downstream (5’–3’) | Product Size（bp） |
| --- | --- | --- | --- |
| *GAPDH* | AAGTTCAACGGCACAGTCAAGG | ACGCCAGTAGACTCCACGACAT | 140 |
| *p75NTR* | CCTCATTCCTGTCTATTGCTCCA | GCGCCTTGTTTATTTTGTTTGC | 105 |
| *Bmal1* | CAGAAGCAAACTACAAGCCAAC | CGGTCACATCCTACGACAAAC | 100 |
| *Clock* | CAGTTCTTACAGACATCTCGGTTG | AAAGTGCTCTGTTGTAGTGGAAAG | 89 |
| *Per1* | ATGCACTTTCAGGCTCCAGT | TTGCTTGTATGGCTGCTCTG | 187 |
| *Runx2* | GAACCAAGAAGGCACAGACAGAA | GGCGGGACACCTACTCTCATACT | 113 |
| *Dlx1* | CAGCCCCTACATCAGTTCCG | CTTCTCCGCCTTCCACCAC | 116 |
